# Identification of ZBTB9 as a potential therapeutic target against dysregulation of tumor cells proliferation and a novel biomarker in Liver Hepatocellular Carcinoma

**DOI:** 10.1186/s12967-022-03790-0

**Published:** 2022-12-15

**Authors:** Zhenshan Zhang, Leilei Wu, Juan Li, Jiayan Chen, Qi Yu, Hui Yao, Yaping Xu, Liang Liu

**Affiliations:** 1 Department of Radiation Oncology, Shanghai Pulmonary Hospital, Tongji University School of Medicine, Shanghai, China; 2 Department of Radiation Oncology, Fudan University Shanghai Cancer Center, Shanghai, 200032 People’s Republic of China; 3 Department of Radiation Oncology, Shanghai Proton and Heavy Ion Center, Fudan University Cancer Hospital, Shanghai, China; 4 Shanghai Concord Cancer Center, Shanghai, 200240 China; 5 Department of Radiation Oncology, Shanghai International Medical Center, Shanghai, China

**Keywords:** LIHC, ZBTB9, TCGA, Prognosis, Biomarker

## Abstract

**Background:**

Zinc finger and bric-a-brac/tramtrack/broad (ZBTB) domain-containing proteins have been reported to be associated with many tumors’ development. However, in tumor initiation and progression, the role of ZBTB9, one of the protein family, and its prognostic value were yet to be elucidated in Liver Hepatocellular Carcinoma (LIHC).

**Methods:**

We used R software and online bioinformatics analysis tools such as GEPIA2, cBioPortal, TIMER2, Metascape, UALCAN, STRING, TISIDB, and COSMIC to investigate ZBTB9’s characteristics and function in LIHC, including abnormal expression, carcinogenic role, related signaling pathways and prognostic value. Furthermore, cell experiments (such as formation, wound healing, and transwell assays) and analyses based on clinical samples (such as immunohistochemistry (IHC) and promoter methylation analysis) were conducted to verify pivotal conclusions.

**Results:**

ZBTB9 was overexpressed in LIHC samples compared to adjacent normal tissues. Through the analysis of genomic alteration and promoter hypomethylation, the clinical value and etiology of abnormal expression of ZBTB9 were preliminarily exlpored. Subsequent evidence showed that it could result in tumor progression and poor prognosis via activating cell cycle, DNA repair, MYC, and KRAS-associated signaling pathways as well as rendering immune dysregulation. After the knockdown of ZBTB9, evidently inhibited capacities of tumor cells proliferation and migration were observed. These results together indicated that ZBTB9 could be a promising prognostic biomarker and had the potential value to offer novel therapeutic targets for LIHC treatment.

**Conclusions:**

ZBTB9 was identified as a novel biomarker to predict the prognosis and tumor progression in LIHC, and a promising therapeutic target to invert tumor development.

**Supplementary Information:**

The online version contains supplementary material available at 10.1186/s12967-022-03790-0.

## Background

Liver Hepatocellular carcinoma (LIHC) is one of the most aggressive cancers. Cancer statistics showed 906,000 new liver cancer cases and 830,000 deaths worldwide in 2020, and the type of 75%–85% liver cancer cases was LIHC [1–3]. In the past decades, efforts were paid to improve the comprehension of LIHC, and many molecules were reported to facilitate LIHC initiation and progression. For example, GOLM1 could drive LIHC metastasis by modulating EGFR/RTK cell-surface recycling [2], and Hsp90α-dependent Bclaf1 could promote LIHC proliferation by regulating c-MYC mRNA stability [3]. However, effective biomarkers are still lacking and the mechanisms underlying LIHC remain largely unclear.

Recently, increasing attention has been paid to ZBTB proteins, which are the family proteins of nuclear transcription factors [4]. These genes could participate in the regulation of multiple gene transcription via binding to corresponding cis-regulatory elements [5]. Notably, many members of them have been reported to play important roles in the development of multiple cancers, such as ZBTB27 [6], ZBTB28 [6], ZBTB33 [7], and ZBTB7 [8]. Recently, for LIHC, the associations between ZBTB20 and LIHC progression have been reported in many studies [9, 10]. Given these, the role of ZBTB proteins in LIHC is worth exploring.

ZBTB9 is a member of ZBTB proteins, and the relationship between its expression and tumor development is poorly studied. In the results of preliminary bioinformatic analysis, we found it was overexpressed in LIHC compared to adjacent normal tissues. Based on this, we conducted further analyses and necessary experiments to investigate its characteristics and prognostic value of it in LIHC. In conclusion, the present study aims to confirm the potential capacity of ZBTB9 to be a novel biomarker and therapeutic target, and simultaneously, to provide novel insight into the underlying molecular mechanisms of LIHC.

## Materials and methods

### Data acquisition

Tumor Immune Estimation Resource 2.0 (TIMER2) (http://timer.cistrome.org/) [11] online tools “Exploration” mode was utilized to obtain the different expression landscape of ZBTB9 between tumor and adjacent normal tissues among cancers. The row data was normalized using log2 TPM (Transcripts Per Kilobase of exon model per Million mapped reads) transformation. Additionally, the RNA-seq data of GSE76427 (52 cancerous tissues, 114 paracancerous tissues) and GSE36376 (193 cancerous tissues and 241 paracancerous tissues) were obtained from Gene Expression Omnibus (GEO) (https://www.ncbi.nlm.nih.gov/) database.

The gene expression arrays and clinical information of the Cancer Genome Atlas (TCGA) [12] LIHC cohorts were obtained from UCSC (http://xena.ucsc.edu/) [13] database, and the expression data were rendered the log2(x + 0.001) swift. The prognostic information was obtained from a previous study [12].

By the tool of Gene Expression Profiling Interactive Analysis 2.0 (GEPIA2) (http://gepia2.cancer-pku.cn/) [14], we acquired the top 50 genes which were the most associated with ZBTB9 in LIHC.

STRING (https://cn.string-db.org/) was a powerful online tool for bioinformatic analysis which could analyze the protein–protein interaction network, based on the evidence from experiments and consensus.

A total of 79 pairs of hepatocellular carcinoma tissues with adjacent normal tissue samples were purchased from ServiceBio (Shanghai, China). H-SCORE (H-SCORE = ∑(pi x i) = (percentage of weak intensity × 1) + (percentage of moderate intensity × 2) + (percentage of strong intensity × 3) [15–17]) was utilized to quantitate the protein stain of IHC. In addition, a total of 10 pairs of frozen LIHC tissues with adjacent normal tissue samples were obtained from Fudan University Shanghai Cancer Center between 2015 and 2018 for the promoter methylation and tumor immune microenvironment (TIME) analysis, and ImageJ software[18] with package of IHC Profiler [19] was utilized to quantitate the IHC results. Samples were stored at -80℃. The use of human tissues conformed to the guidelines of the Declaration of Helsinki.

### Diagnostic and prognostic value of ZBTB9

With the pROC package of R (v 3.6.3), the receiver operating characteristic curve (ROC) was depicted to show its diagnostic value.

The Kaplan–Meier survival analysis and cox regression model were performed via the “survminer” and “survival (v 3.2-7)” packages of R, and the P-value, 95% confidence intervals (CI), and hazard ratio (HR) were also computed.

### Genetic alteration analysis

cBioPortal database (https://www.cbioportal.org/) [20, 21] was used to estimate the genetic alteration landscape of ZBTB9 among cancers with the data of TCGA PanCancer Atlas Studies. In addition, the information of alteration regions was obtained with the "Mutations" module.

The Catalogue of Somatic Mutations (COSMIC) (http://www.sanger.ac.uk/cosmic/) [22] is another public resource that offers information on somatically acquired mutations among human cancers for input genes.

### DNA promoter methylation analysis with the public database

Through UALCAN (http://ualcan.path.uab.edu/analysisprot.html) [23], we acquired the box plots of ZBTB9 promoter methylation levels under different clinical characteristics based on the TCGA database.

### Methylation-specific polymerase chain reaction (MSP) analysis

MSP analysis was performed using primers for methylated or unmethylated DNA designed using MethPrimer. Briefly, 2 μl of bisulfite-treated genomic DNA was amplified using TaKaRa EpiTaq HS (Takara Bio). The cycles setting is 95 °C for 5 min, followed by 35 cycles of 94 °C for 20 s, 60 °C for 30 s, and 72 °C for 20 s, followed by a single cycle of 72 °C for 5 min. then, the PCR products were analyzed using 2% agarose gel electrophoresis. The results were quantitated via ImageJ software [18]. All measurements were performed in triplicate. The primer sequences used for MSP are listed below.

ZBTB9-M-F: 5′-GGTGTTATATTTAATTATCGGGAAAC-3′

ZBTB9-M-R: 5′-AACTACTACAAACGAAAAAAACGAT-3′

ZBTB9-UM-F: 5′-GTTATATTTAATTATTGGGAAATGT-3′

ZBTB9-UM-R: 5′-AACTACTACAAACAAAAAAAACAAT-3′.

### Analysis of drug treatment and ZBTB9 expression

We logged in CellMiner online database (https://discover.nci.nih.gov/cellminer/home.do) [24, 25] to get the correlation between pharmacological treatment efficacy and gene expression level.

### Immunocytes infiltration analysis

By the TISIDB database (http://cis.Hku.hk/TISIDB/) [26], we analyzed the correlation between ZBTB9 and TIME, including tumor-infiltration lymphocytes, immunocyte co-inhibitors, and co-stimulators in this platform, which were plotted with heat maps and scatter graphs. TISIDB is a web portal for tumor and immune system interaction, which integrates multiple heterogeneous data types, including the literature mining results from the PubMed database, high throughput screening data, exome, and RNA sequencing data sets of patient cohorts with immunotherapy, genomics, transcriptomics and clinical information of 30 cancer types from The Cancer Genome Atlas (TCGA) and public databases, including UniProt, GO, DrugBank, etc.

IHC of ZBTB9 (antibody: NBPY-92611, Novus), CD8A (antibody: 8M4102, BOSTER), and FOXP3 (antibody: PB0043, BOSTER) proteins in the 20 samples containing 10 tumor and 10 adjacent normal tissues was conducted to explore the relationship between ZBTB9 and TIME.

### Gene set enrichment analysis

The Gene set enrichment analysis (GSEA) [27] method was applied to study the potential mechanisms of ZBTB9 behind the initiation and progression of LIHC via clusterProfiler package (v 3.14.3) [28]. We obtained the gene collections (h.all.v7.2.symbols.gmt) from Molecular Signatures Database [29]. According to the median expression level of ZBTB9, samples were divided into high and low groups. Via comparing the differences in genes level between the two groups, the upregulated and downregulated genes were identified. Then the genes were enriched based on the database of Hallmark gene sets. Gene sets with |normalized enrichment score (NES)|> 1, and false discovery rate (FDR) < 0.05 were considered significant results.

### Associated genes’ function analysis

Gene Ontology (GO) and Kyoto Encyclopedia of Gene and Genomes (KEGG) pathway analysis were utilized to investigate the function of the gene set with R package clusterProfiler (3.14.3) and org.Hs.eg.db (3.10.0). GO analysis is a common method used for annotating genes and gene products and for identifying molecular function (MF), biological process (BP), and cellular components (CC). KEGG is a collection of databases for systematic analysis of gene functions and associating related gene sets with their pathways. In addition, we also utilized the Metascape (http://metascape.org) tool [30] to analyze the functions of the related genes of ZBTB9 in LIHC.

With GSCALite (https://www.editorialmanager.com/jtrm/default1.aspx) [31], the association of activation signaling pathways and ZBTB9-related genes was also analyzed.

### Cell culture

HepG2 cells were obtained from the Chinese Academy of Sciences Cell Bank and cultured in Dulbecco’s modified Eagle’s medium (DMEM) (Gibco Thermo Fisher Scientific, Inc. USA), supplemented with 10% fetal calf serum (FBS) (Gibco Thermo Fisher Scientific, Inc. USA), 100 U/ml penicillin, and 100 μg/ml streptomycin (Solarbio, Beijing, China), and cultured at 37 °C in a 5% CO2 humid atmosphere.

### ZBTB9 knockdown

To block ZBTB9 expression in HepG2 cells, two small interferings (si) RNAs against ZBTB9 (siZBTB9-1 and siZBTB9-2) and one negative control (siCtrl) RNA were designed and synthesized by Sangon Biotech Co., Ltd. For transfection, cells were seeded into 6-well plates at a concentration of 1 × 10^5 cells/well and maintained at 37 °C until 70% confluent. Then, cells were transfected with siRNAs or siCtrl using Lipofectamine® 3000 (Invitrogen; Thermo Fisher Scientific, Inc.) following the manufacturer’s protocols. The antisense sequences of the three siRNAs were shown as followed:

siZBTB9-1: 5′-TTTCTGAGCACTGATCTACTA-3′.

siZBTB9-2: 5′-TAACACTGCTTTATGAGCCCT-3′.

siCtrl: 5′-TTCTCCGAACGTGTCACGTTT-3′.

### Knockdown efficiency evaluation by RT-qPCR and western blot (WB)

Relative expression of ZBTB9 in HepG2 cells was evaluated via RT-qPCR. We extracted total RNA from cells using TRIzol reagent (Invitrogen, USA). cDNA was synthesized using the RevertAid First Strand cDNA Synthesis kit (Promega Corporation, USA) following the manufacturer's instructions. RT-qPCR was conducted using the iQ™ SYBR Green Supermix (Bio-Rad, USA). The following primers were used for qPCR:

ZBTB9-F: 5′-TCGGCGGGAAGGACAATC-3′,

ZBTB9-R: 5′-TAGACTCCACGCGGGATGAA-3′,

GAPDH-F: 5′-ACAACTTTGGTATCGTGGAAGG-3′

GAPDH-R: 5′-GCCATCACGCCACAGTTTC-3′.

The 2-ΔΔCt method was conducted to calculate the relative expression levels of targets.

For WB assay, the molecular weight of ZBTB9 and GAPDH are 50.602 kDa and 36.053 kDa, respectively, and total proteins were extracted from HepG2 cells using RIPA lysis buffer and subsequently were quantified using the Bradford method (Bio-Rad, Hercules CA, USA). Twenty micrograms of protein were loaded and separated by polyacrylamide gel electrophoresis and then transferred to PVDF membranes (Bio-Rad). After blocking with 5% BSA, membranes were incubated with specific antibodies for ZBTB9 (1:1000, Abcam), and GAPDH (1:1000, Abcam) at 4 °C overnight. On the next day, the membranes were incubated with horseradish peroxidase-conjugated secondary antibody (1:3000) for 2 h at room temperature. The Quantity One software package (Bio-Rad, USA) was used for the quantitation of signal intensities.

### Wound healing assay

HepG2 cells were seeded into a 6-well plate. When the cells' confluence reached 80%, we scratched the monolayer cells with a sterile micropipette tip. The floating cells were then washed with PBS. Wound healing within the scrape line was observed at 0 h and 16 h. Triplicate wells for each condition were examined.

### Cell cycle analysis

Flow cytometry analysis was performed to estimate the effects of ZBTB9 knockdown on the HepG2 cell cycle. After 48 h transfection with siZBTB9 or siCtrl, HepG2 cells were trypsinized and centrifuged for 10 min at 2000 rpm, followed by three washes with pre-cold PBS. Then, cells were fixed with 70% ethanol at -20˚C overnight, and cell cycle distribution was analyzed using the cell cycle kit (BestBio) and a Beckman Coulter FACSCalibur flow cytometer (Beckman Coulter, Inc.).

### Colony formation assay

The effects of ZBTB9 knockdown on LIHC cell proliferation were assessed by colony formation assay. After 48 h transfection with siZBTB9 or siCtrl, HepG2 cells were seeded into 6-well plates at a density of 1000 cells/well, followed by 1 week of incubation at 37 °C with 5% CO2. Subsequently, colonies were fixed using methanol for 1 h and stained with Giemsa solution for 20 min. The number of colonies (> 50 cells) was manually calculated under a microscope, and triplicate wells were evaluated for each group.

### Transwell assay

Transwell assay using 24-well plates with an 8-µm pore chamber (Corning, Inc.), and Matrigel matrix was diluted 1:8 to coat the upper side of the membrane at the bottom of the transwell chamber. Cells were first cultured in 2% FBS-DMEM medium for 12 h for starvation treatment and suspended in FBS-free DMEM medium, then were added to the upper chamber (1 × 10^5 cells/well), respectively. Meanwhile, DEME medium with 10% FBS was added to the lower compartment. The plates were incubated for 72 h in the incubator. After incubation, cells that migrated to the lower surface of the filter membrane were fixed with 4% paraformaldehyde and stained with 0.5% crystal violet. Cells remaining on the upper surface of the filter membrane were gently scraped off with a cotton swab. The lower surfaces were captured by the inverted microscope, and the counting was repeated three times.

### Statistical analysis

Parts of packages of R were introduced as previously reported, ggplot2 package and Graphpad prism 9.0 software was utilized to visualize the results of statistical analyses, besides, the chi-squared test, Student’s test, Wilcoxon test, and Kruskal–Wallis tests were used appropriately to calculate the significance of differences in data between groups.

## Results

### The abnormal expression of ZBTB9 in LIHC

**Fig. 1:**
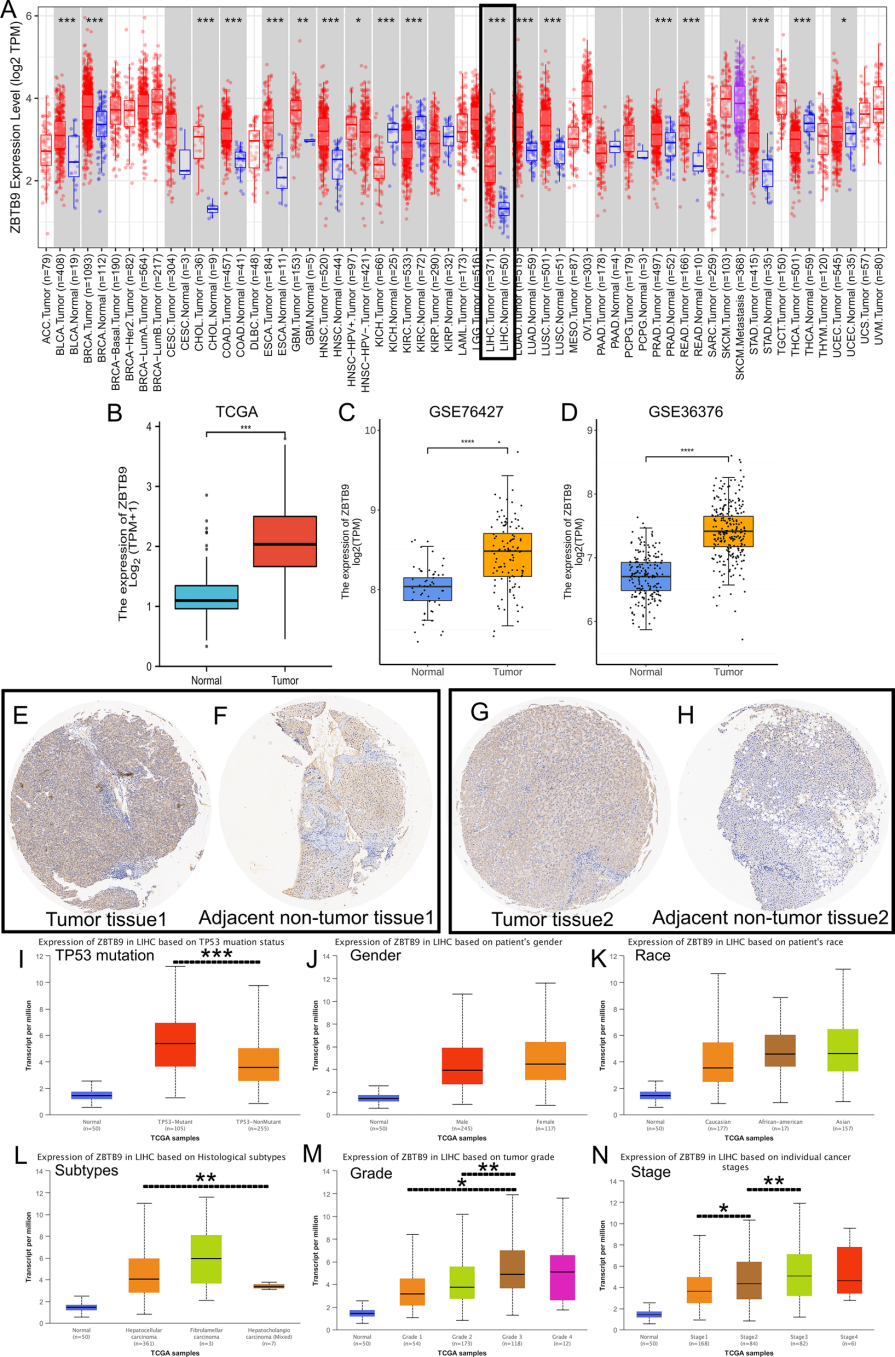
ZBTB9 expression landscape. The expression distribution of ZBTB9 among tumor tissues and normal tissues with TIMER2 (**A**). The expression level of the ZBTB9 gene was overexpressed in TCGA LIHC patients’ database (374 cancerous tissues and 50 paracancerous tissues) (**B**), GSE76427 (52 cancerous tissues and 114 paracancerous tissues) (**C**) and GSE36376 (193 cancerous tissues and 241 paracancerous tissues) (**D**). IHC of LIHC samples, cancerous tissue (**E**, **G**), paracancerous tissue (**F**, **H**). The higher expression level of the ZBTB9 gene was detected in the subgroups of female, TP53 mutation, fibrolamellar carcinoma, higher tumor grade, and higher individual cancer stages (**I**–**N**). (*p < 0.05; **p < 0.01; ***p < 0.001)

Genes with aberrant expression in tumor samples were considered to probably participate in tumor initiation and development. Herein, we primarily found that ZBTB9 was significantly upregulated in 15 types of tumors compared to corresponding normal tissues with TIMER2 pan-cancer data, including BLCA, BRCA, CHOL, COAD, ESCA, GBM, HNSC, HNSC-HPV (−), LIHC, LUAD, LUSC, PRAD, READ, STAD and UCEC (all P < 0.05) (Fig. 1A), and the specific information of these cancer types were described in Table 1. Further, with TCGA LIHC data, it showed that ZBTB9 was significantly overexpressed in LIHC samples compared to adjacent normal tissues plus GTEx normal tissues with the P < 0.001 (Fig. 1B). Then based on GSE76427 and GSE36376 of GEO database, evident upregulation of ZBTB9 was also detected in LIHC tumor tissues compared to normal tissues (Fig. 1C, D). To further confirm these findings, we conducted ZBTB9 IHC between the tumor and paired adjacent normal tissues, results also demonstrated that ZBTB9 protein was significantly higher in tumor samples (Fig. 1E–H).

**Table 1:** Abbreviations of tumor types

BLCA	Bladder urothelial carcinoma
BRCA	Breast invasive carcinoma
CHOL	Cholangiocarcinoma
COAD	Colon adenocarcinoma
ESCA	Colon adenocarcinoma
GBM	Glioblastoma multiforme
GBMLGG	Glioma
HNSC	Glioblastoma multiforme
LAML	Acute myeloid leukemia
LIHC	Glioblastoma multiforme
LUAD	Lung adenocarcinoma
LUSC	Lung squamous cell carcinoma
PRAD	Prostate adenocarcinoma
READ	Rectum adenocarcinoma
SARC	Sarcoma
SKCM	Skin cutaneous melanoma
STAD	Stomach adenocarcinoma
UCEC	Uterine corpus endometrial carcinoma

### The correlation between ZBTB9 level and clinical pathologic characteristics

The correlations between ZBTB9 expression levels and different clinical characteristics were analyzed via the UALCAN database, which indicated that higher ZBTB9 expression was significantly associated with higher individual stages and tumor grade (Fig. 1M, N). The association of H-SCORE and clinical features based on 79 samples of tissue microarray was offered in Table 2, which confirmed that high ZBTB9 protein in tumor samples was related to high T stages. The above findings indicated that ZBTB9 was overexpressed in many cancers including LIHC, and showing significant association with tumor grade and stage, hence, its role in tumorigenesis and prognosis was worth further investigating.

**Table 2:** Correlation between clinical features and H-SCORE of IHC based on the tissue microarray

Characteristic	Low scores (n = 39)	High scores (n = 40)	p. value
	No. (%)	No. (%)	
Gender, n (%)			0.511
Female	8 (10.1%)	5 (6.3%)	
Male	31 (39.2%)	35 (44.3%)	
T stage, n (%)			**0.039**
T1a	13 (16.5%)	5 (6.3%)	
T1b	12 (15.2%)	12 (15.2%)	
T2	13 (16.5%)	16 (20.3%)	
T3	1 (1.3%)	7 (8.9%)	
N stage, n (%)			0.615
N0	37 (46.8%)	39 (49.4%)	
N1	2 (2.5%)	1 (1.3%)	
M status, n (%)			0.066
M0	37 (46.8%)	31 (39.2%)	
M1	2 (2.5%)	8 (10.1%)	
M2	0 (0%)	1 (1.3%)	
HBsAg, n (%)			0.953
Negative	9 (11.4%)	8 (10.1%)	
Positive	30 (38%)	32 (40.5%)	
HBcAb, n (%)			1.000
Negative	7 (8.9%)	7 (8.9%)	
Positive	32 (40.5%)	33 (41.8%)	
Age, mean ± SD	53.82 ± 9.37	50.38 ± 12.66	0.173

### Genetic alterations and promoter methylation analysis of ZBTB9

The analysis of the genetic alteration and promoter methylation of ZBTB9 could contribute to the comprehension of the etiology for its abnormal expression and the correlation between these alterations and clinical pathologic characteristics.

**Fig. 2:**
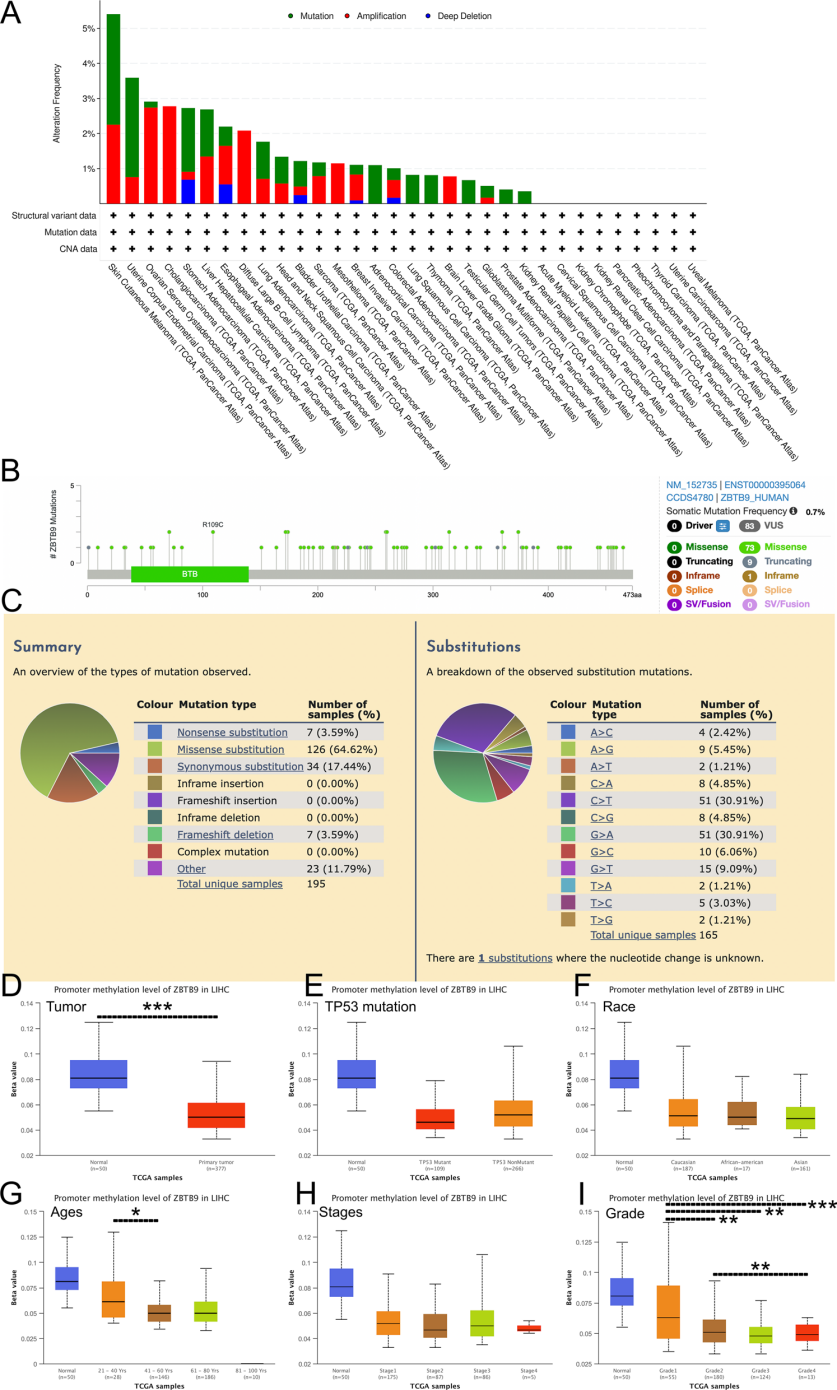
ZBTB9 genetic alteration and promoter methylation analysis. ZBTB9 alteration in pan-cancers (**A**). Types of ZBTB9 mutation in pan-cancers (**B**). Types and substitution of ZBTB9 mutation in pan-cancers (**C**). Correlation between promoter methylation level of ZBTB9 and clinical characteristics (D-I). (*p < 0.05; **p < 0.01; ***p < 0.001)

ZBTB9 genetic mutations and alterations landscape were investigated via cBioPortal (Fig. 2A) database. According to the results, the main type of its genetic alterations was "mutation", which was observed in the bulk of TCGA cancers, and "amplification" was the second most common. Moreover, the frequency of ZBTB9 alteration in LIHC patients was 2.69% in 372 cases, comprised of “mutation” and “amplification”. The “deep deletion” type in cancers was rare. We further explored the specific mutation type and site of ZBTB9 among cancers (Fig. 2B). Then, with the COSMIC online tool, the overview of the types of mutation was also observed. The primary mutation type was missense substitution (64.62%), which was similar to the result of cBioProtal, and the primary substitution mutation types were G > A (30.91%) and C > T (30.91%) (Fig. 2C).

Promoter methylation analysis was conducted via UALCAN, and we analyzed the ZBTB9 methylation levels, which showed that the promoter methylation level of ZBTB9 in LIHC tumor tissues was significantly lower than that in adjacent normal tissues (Fig. 2D). In terms of the individual cancer stage and tumor grade, there was a trend that lower promoter methylation came with higher tumor stage and tumor grade, which was more significant in tumor grade (Fig. 2H and I). Also, the promoter hypomethylation of ZBTB9 was confirmed with our independent samples. According to the results of agarose gel electrophoresis (Additional file 1: Fig. S1A), it was obvious that in most samples, the promoter methylations of ZBTB9 were significantly lower in tumor samples than paired adjacent normal tissues (P = 0.033, Additional file 1: Fig. S1B) (the odd labels are adjacent normal tissues and even labels are tumor tissues).

These findings demonstrated that the abnormal expression of ZBTB9 in certain cancers could be caused via genetic alterations and promoter hypomethylation, which would contribute to the comprehensive cognition of LIHC tumorigenesis and guidance for potential treatment strategy.

### Prognostic and diagnostic value analysis

**Fig. 3:**
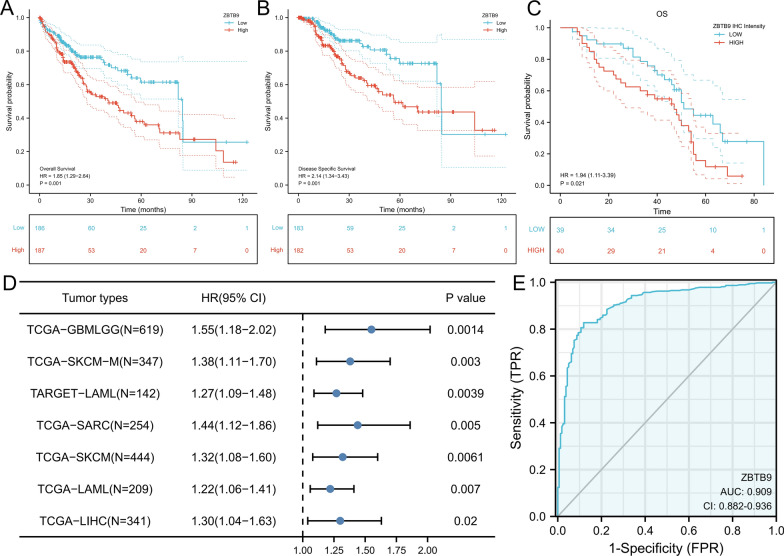
High expression of ZBTB9 indicated poor survival in patients with LIHC. OS analysis in LIHC (**A**), DSS analysis in LIHC (**B**), and OS analysis with 79 samples (**C**). The forest plot showed the ZBTB9 hazard rate in different cancers (**D**). Diagnostic ROC analysis with the AUC of ZBTB9 in LIHC (**E**) (*p < 0.05; **p < 0.01; ***p < 0.001)

The OS and disease special survival (DSS) analysis based on LIHC cohorts [12] were performed via R software. The results showed that high expression of ZBTB9 was associated with evidently shorter OS (HR = 1.85, 95% CI 1.29–2.64, P = 0.001) and shorter DSS (HR = 2.14, 95% CI 1.34–3.43, P = 0.001) (Fig. 3A, B). The survival analysis based on the IHC H-SCOREs of our independent 79 LIHC samples also indicated ZBTB9 was a risk factor in their OS (Fig. 3C). The TCGA LIHC clinical characteristics under different ZBTB9 expression levels were shown in Table 3.

**Table 3:** Relationship between ZBTB9 expression level and clinicopathological variables in LIHC patients

Characteristic	Low expression of ZBTB9 (n = 187) No. (%)	High expression of ZBTB9 (n = 187) No. (%)	p. value
T stage			**0.007**
T1	107 (57.22%)	76 (40.64%)	
T2	41 (21.93%)	54 (28.88%)	
T3	30 (16.04%)	50 (26.74%)	
T4	7 (3.74%)	6 (3.21%)	
Unknown	2 (1.07%)	1 (0.53%)	
N stage			1.000
N0	126 (67.38%)	128 (68.45%)	
N1	2 (1.07%)	2 (1.07%)	
Unknown	59 (31.55%)	57 (30.48%)	
M stage			1.000
M0	132 (70.59%)	136 (72.73%)	
M1	2 (1.07%)	2 (1.07%)	
Unknown	59 (28.34%)	49 (26.20%)	
PFI event			0.301
Alive	101 (54.01%)	90 (48.13%)	
Dead	86 (45.99%)	97 (51.87%)	
DSS event			** < 0.001**
Alive	158 (84.49%)	129 (68.98%)	
Dead	26 (13.90%)	53 (28.34%)	
Unknown	3 (1.60%)	5 (2.67%)	
OS event			** < 0.001**
Alive	140 (74.87%)	104 (55.61%)	
Dead	47 (25.13%)	83 (44.39%)	
Vascular invasion			0.399
No	112 (59.89%)	96 (51.34%)	
Yes	53 (28.34%)	57 (30.48%)	
Unknown	22 (11.76%)	34 (18.18%)	
Fibrosis ishak score			0.341
0	39 (20.86%)	36 (19.25%)	
1/2	16 (8.56%)	15 (8.02%)	
3/4	14 (7.49%)	14 (7.49%)	
5/6	52 (27.81%)	29 (15.51%)	
Unknown	66 (35.29%)	93 (49.73%)	
Child–Pugh grade			0.138
A	116 (62.03%)	103 (55.08%)	
B	15 (8.02%)	6 (3.21%)	
C	1 (0.53%)	0 (0.00%)	
Unknown	55 (29.41%)	78 (41.71%)	
Prothrombin time			0.849
≤ 4	108 (57.75%)	100 (53.48%)	
> 4	48 (25.67%)	41 (21.93%)	
Unknown	31 (16.58%)	46 (24.60%)	
Albumin(g/dl)			1.000
≤ 3.5	36 (19.25%)	33 (17.65%)	
> 3.5	121 (64.71%)	110 (58.82%)	
Unknown	30 (16.04%)	44 (23.53%)	
AFP (ng/ml)			**0.036**
≤ 400	120 (64.17%)	95 (50.80%)	
> 400	26 (13.90%)	39 (20.86%)	
Unknown	41 (21.93%)	53 (28.34%)	
Adjacent hepatic tissue inflammation			0.696
None	66 (35.29%)	52 (27.81%)	
Mild	51 (27.27%)	50 (26.74%)	
Severe	9 (4.81%)	9 (4.81%)	
Unknown	61 (32.62%)	76 (40.64%)	
Histologic grade			0.198
G1	33 (17.65%)	22 (11.76%)	
G2	82 (43.85%)	96 (51.34%)	
G3	61 (32.62%)	63 (33.69%)	
G4	8 (4.28%)	4(2.14%)	
Unknown	3 (1.60%)	2 (1.07%)	
Residual tumor			0.179
R0	167 (89.30%)	160 (85.56%)	
R1	6 (3.21%)	11 (5.88%)	
R2	0 (0.00%)	1 (0.53%)	
Unknown	14 (7.49%)	15 (8.02%)	
BMI			0.168
≤ 25	83 (44.39%)	94 (50.27%)	
> 25	88 (47.06%)	72 (38.50%)	
Unknown	16 (8.56%)	21 (11.23%)	
Heigh			0.062
≤ 170	93 (49.73%)	108 (57.75%)	
> 170	80 (42.78%)	60 (32.09%)	
Unknown	14 (7.49%)	19 (10.16%)	
Weight			0.194
≤ 70	86 (45.99%)	98 (52.41%)	
> 70	88 (47.06%)	74 (39.57%)	
Unknown	13 (6.95%)	15 (8.02%)	
Age			0.961
≤ 60	89 (47.59%)	88 (47.06%)	
> 60	97 (51.87%)	99 (52.94%)	
Unknown	1 (0.53%)	0 (0.00%)	
Race			0.571
Asian	75 (40.11%)	85 (45.45%)	
Black or African. American	9 (4.81%)	8 (4.28%)	
White	97 (51.87%)	88 (47.06%)	
Unknown	81(43.32%)	91 (48.66%)	
Gender			0.269
Female	55 (29.41%)	66 (35.29%)	
Male	132 (70.59%)	121 (64.71%)	
Tumor status			**0.010**
Tumor free	115 (61.50%)	87 (46.52%)	
With tumor	65 (34.76%)	88 (47.06%)	
Unknown	7 (3.74%)	12 (6.42%)	
Pathologic stage			**0.024**
I	102 (54.55%)	71 (37.97%)	
II	38 (20.32%)	49 (26.20%)	
III	36 (19.25%)	49 (26.20%)	
IV	3 (1.60%)	2 (1.07%)	
Unknown	8 (4.28%)	16 (8.56%)	
Age, median (IQR)	61 (54–68, 32.62%)	61 (51–70, 32.62%)	0.944
Unknown	126 (67.38%)	126 (67.38%)	

Furthermore, with the coxph function of the survival package, cox proportional hazard regression analysis was rendered to analyze the HR of ZBTB9 among cancers (only the significant results were given), which indicated that ZBTB9 was a significant risk factor in many cancers, including GBMLGG (HR = 1.55, 95% CI 1.18–2.02, P = 0.0014), SKCM-M (HR = 1.38, 95% CI 1.11–1.70, P = 0.003), LAML (HR = 1.27, 95% CI 1.11–1.07, P = 0.003), SARC (HR = 1.44, 95% CI 1.12–1.86, P = 0.005), SKCM (HR = 1.32, 95% CI 1.08–1.60, P = 0.0061), LAML (HR = 1.22, 95% CI 1.06–1.41, P = 0.007) and LIHC (HR = 1.30 95% CI 1.04–1.63, P = 0.02) (Fig. 3D), and the specific information of these cancer types were described in Table 1. These results showed that a relatively high level of ZBTB9 was often related to a worse outcome of tumor patients. In addition, the area under the diagnostic receiver operating curve (ROC) was 0.909 (95% CI 0.882–0.936) (Fig. 3E).

Subsequently, univariate cox regression analysis showed that ZBTB9 level (P < 0.001), pathologic stage (P < 0.001), T stage (P < 0.001) M stage (P = 0.017), and tumor status (P < 0.001) were all significantly correlated with OS. Besides, ZBTB9 expression was also significantly correlated with OS in multivariate cox regression analysis (P = 0.014), suggesting that ZBTB9 was an independent prognostic factor in LIHC patients (Table 4).

**Table 4:** Univariable and multivariable analysis of ZBTB9 associated with OS, and the unknown data in each group were excluded before multivariate analysis

Characteristics	Total (N)	Univariate analysis		Multivariate analysis	
		Hazard ratio (95% CI)	P. value	Hazard ratio (95% CI)	P. value
T stage	370				
T1 & T2	277	1.000		1.000	
T3 & T4	93	2.598 (1.826–3.697)	** < 0.001**	1.531 (0.208–11.249)	0.676
N stage	258				
N0	254	1.000			
N1	4	2.029 (0.497–8.281)	0.324		
M stage	272				
M0	268	1.000			
M1	4	4.077 (1.281–12.973)	**0.017**	1.262 (0.301–5.302)	0.750
Gender	373				
Female	121	1.000			
Male	252	0.793 (0.557–1.130)	0.200		
Pathologic stage	349				
I & II	259	1.000		1.000	
III & IV	90	2.504 (1.727–3.631)	** < 0.001**	1.610 (0.220–11.798)	0.639
Race	361				
Asian	159	1.000			
White	185	1.323 (0.909–1.928)	0.144		
Black or African American	17	1.585 (0.675–3.725)	0.290		
Residual tumor	344				
R0	326	1.000			
R1&R2	18	1.604 (0.812–3.169)	0.174		
BMI	336				
≤ 25	177	1.000			
$>$ 25	159	0.798 (0.550–1.158)	0.235		
AFP (ng/ml)	279				
≤ 400	215	1.000			
> 400	64	1.075 (0.658–1.759)	0.772		
Vascular invasion	317				
No	208	1.000			
Yes	109	1.344 (0.887–2.035)	0.163		
Histologic grade	368				
G1	55	1.000			
G2	178	1.162 (0.686–1.969)	0.576		
G3	123	1.185 (0.683–2.057)	0.545		
G4	12	1.681 (0.621–4.549)	0.307		
Age	373				
≤ 60	177	1.000			
> 60	196	1.205 (0.850–1.708)	0.295		
Tumor status	354				
Tumor free	202	1.000		1.000	
With tumor	152	2.317 (1.590–3.376)	**< 0.001**	1.854 (1.156–2.975)	**0.010**
ZBTB9	373				
Low	186	1.000		1.000	
High	187	1.848 (1.291–2.644)	**< 0.001**	1.779 (1.125–2.812)	**0.014**

These results further indicated that the expression level of ZBTB9 was prone to be associated with shorter survival of LIHC patients, and also verified that ZBTB9 participated in the initiation and development of LIHC.

### Survival analysis in different subtypes

**Fig. 4:**
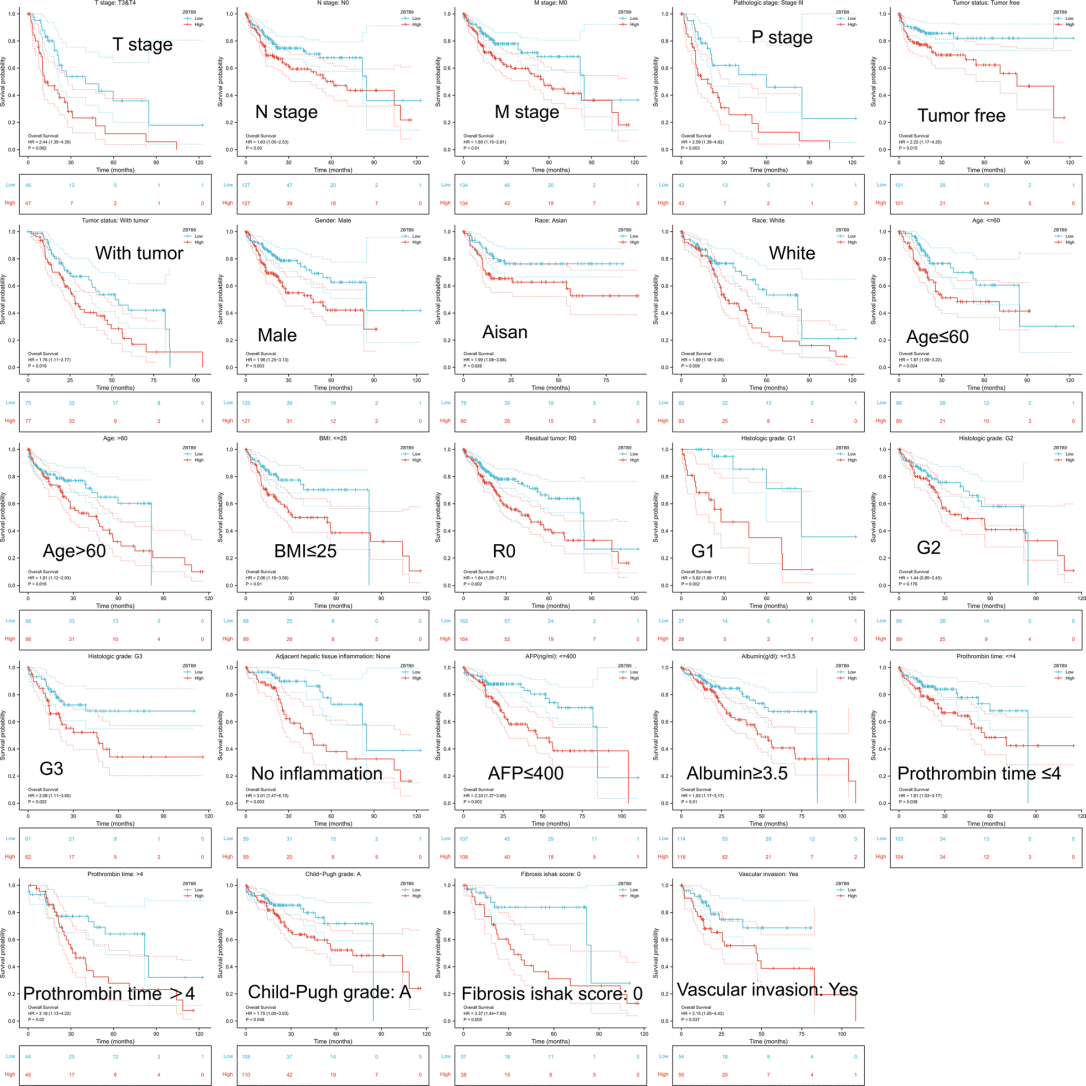
Survival analysis of ZBTB9 in different clinical subtypes, and pharmacologic response. Kaplan–Meier curve analysis was used to investigate the relationship between ZBTB9 expression and clinical variables (*p < 0.05; **p < 0.01; ***p < 0.001)

The Kaplan–Meier survival curves analysis was also performed to evaluate the prognostic value of ZBTB9 under different clinical characteristics. The results indicated that the high ZBTB9 expression was an evident risk factor in 24 subtypes, including the groups of T stage: T3 plus T4 (HR = 2.44), N stage: N0 (HR = 1.63), M stage: M0 (HR = 1.80), pathologic stage: Stage III (HR = 2.59), tumor status: With tumor (HR = 1.76), gender: male (HR = 1.98), race: Asian (HR = 1.99), Race: white (HR = 1.89), age: ≤ 60 (HR = 1.87), age: ≥ 60 (HR = 1.81), BMI: ≤ 25 (HR = 2.06), residual tumor: R0 (HR = 1.84), histologic grade: G1 (HR = 5.82), histologic grade: G2 (HR = 1.44), histologic grade: G3 (HR = 2.08), adjacent hepatic tissue inflammation: None (HR = 3.01), AFP: ≤ 400 ng/ml (HR = 2.33), Albumin: ≥ 3.5 g/dl (HR = 1.93), Prothrombin time: ≤ 4 (HR = 1.81), Prothrombin time: > 4 (HR = 2.19), Child–Pugh grade: A (HR = 1.75), Fibrosis ishak score: 0 (HR = 3.37), Vascular invasion: Yes (HR = 2.15), with all P values < 0.05 (Fig. 4).

### Correlation between ZBTB9 expression and TIME

As we have known that immunity is an essential part of tumor initiation, growth, and treatment, and the infiltrating lymphocytes’ function could be mainly influenced by immunomodulators in TIME. Herein, to evaluate the relationship between ZBTB9 and TIME is necessary.

**Fig. 5:**
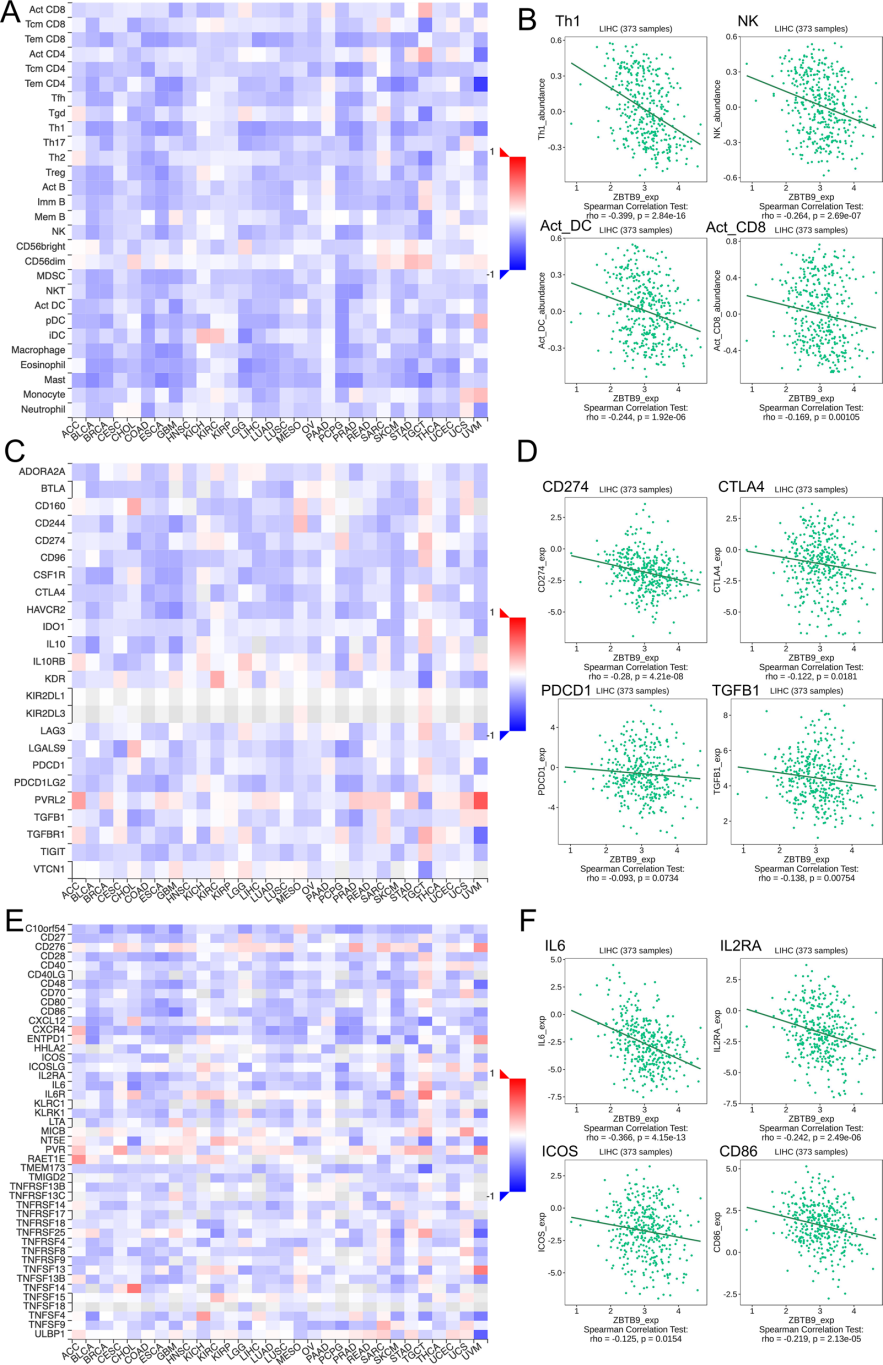
Correlation analysis of ZBTB9 level and immunity. Relations with tumor-infiltrating lymphocytes in cancers (**A**), and top 4 results in LIHC (**B**). Relations with immune inhibitors in cancers (**C**), and top 4 correlation results (**D**). Relations with stimulators in cancers (**E**). Relations with immune and top 4 results in LIHC (**F**) (*p < 0.05; **p < 0.01; ***p < 0.001)

Therefore, we conducted the analyses to explore the correlation of ZBTB9 expression between lymphocytes infiltration, chemokines, immune checkpoint inhibitors, and immune checkpoint stimulators in LIHC, and for each part, 4 main results were provided. It demonstrated that ZBTB9 was negatively associated with the infiltration of mainly immune cells (Fig. 5A, B), including Th1 cells, (Cor = − 0.399, P < 0.001), NK cells (Cor = − 0.264, P < 0.001), activated dendritic cells (DCs) (Cor = − 0.244, P < 0.001), activated CD 8+ T cell (Cor = − 0.169, P = 0.001), and also negatively associated with inhibitors (Fig. 5C, D), such as CD274 (Cor = − 0.28, P < 0.001). The correlation between ZBTB9 expression and stimulators were still mainly negative (Fig. 5E, F), IL6 (Cor = − 0.366, P < 0.002), IL2RA (Cor = − 0.242, P < 0.001), ICOS (Cor = − 0.125, P < 0.002) and CD86 (Cor = − 0.219, P < 0.001).

Further, the IHC of TIME showed that, in two tumor samples, the patient with a relatively higher level of ZBTB9 (sample #2, Additional file 2: Fig. S2B) have a relatively low staining intensity of CD8A (although the IHC result of CD8A in sample #2 was positive too, the proportion of high positive intensity was still lower than sample #1, Additional file 2: Fig. S2D), and FOXP3 (Additional file 2: Fig. S2F), which was basically consistent with previous conclusions, a negative correlation between ZBTB9 level and CD8+ T and Tregs in TIME.

In conclusion, it seems that ZBTB9 was prone to associate with “Cold” TIME, which demonstrated that patients with high ZBTB9 levels might have less infiltration of immune effective cells in the tumor site, and perhaps has a low response to immune checkpoint inhibitors treatment, such as anti-PD1 and anti-PDL1.

### Gene set enrichment analysis

**Fig. 6:**
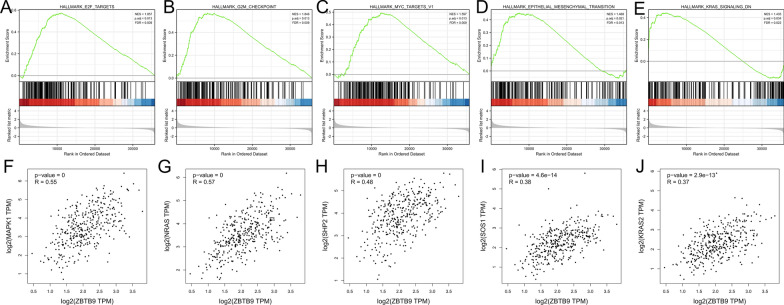
Gene set enrichment analysis (GSEA) between the high ZBTB9 expression group and low ZBTB9 expression group. **A**–**E** GSEA analysis with MsigDB of hallmark. **F**–**J** Correlation analysis between ZBTB9 and KRAS signaling pathways core genes (*p < 0.05; **p < 0.01; ***p < 0.001)

The enrichment results based on Hallmarks gene sets demonstrated that high ZBTB9 was related to the activation of certain signaling pathways such as E2F targets (NES = 1.857, P.adj value = 0.013), G2M checkpoint (NES = 1.846, P.adj value = 0.013), MYC TARGETS V1 (NES = 1.597, P.adj value = 0.013), EPITHELIAL MESENCHYMAL TRANSITION (NES = 1.468, P.adj value = 0.021), KRAS SIGNALING DN (NES = 1.435, P.adj value = 0.034), (Fig. 6A–E). These pathways might be the potential downstream mechanisms following the ZBTB9 overexpression. Subsequent correlation analysis showed that ZBTB9 expression was evidently related to the core molecules of KRAS signaling pathways (Fig. 6F–J), suggesting the potential value of ZBTB9 as a novel therapeutic target against this pathway in LIHC.

### Drug-efficacy analysis

**Fig. 7:**
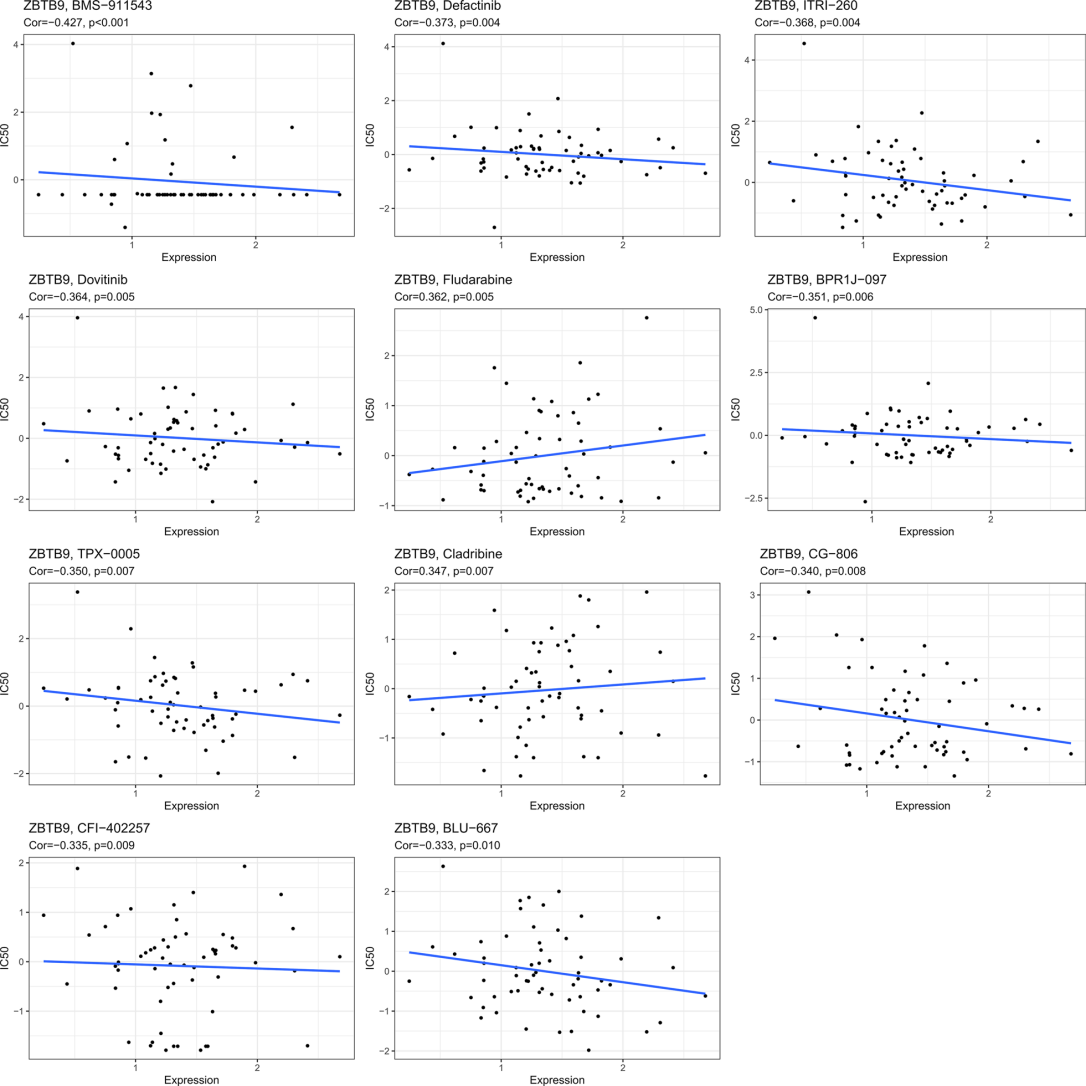
Scatter plots showed the correlation between ZBTB9 expression and IC50 of drugs (*p < 0.05; **p < 0.01; ***p < 0.001)

The data of pharmacologic efficacy and mRNAs level were obtained from CellMiner, then the correlation between drugs half maximal inhibitory concentration (IC50) and ZBTB9 gene expression level was analyzed, which showed that ZBTB9 level was significantly negatively related to the IC50 of BMS-911543 (Cor = − 0.427, P < 0.001), Defactinib (Cor = − 0.373, P = 0.004), ITRI-260 (Cor = − 0.368, P = 0.004), Dovitinib (Cor = − 0.364, P = 0.005), BPR1J-097 (Cor = − 0.351, P = 0.006), TPX-0005 (Cor = − 0.350, P = 0.007), CG-806 (Cor = − 0.340, P = 0.008), CFI-402257 (Cor = − 0.335, P = 0.009) and BLU-667 (Cor = − 0.333, P = 0.010), and positively related with Fludarabine (Cor = 0.362, P = 0.005), Cladribine (Cor = 0.347, P = 0.007) (Fig. 7), which help to optimize the guidance of personalized treatment for ZBTB9 high expression LIHC patients.

### Identification of ZBTB9-related genes and associated biological function analysis

**Fig. 8:**
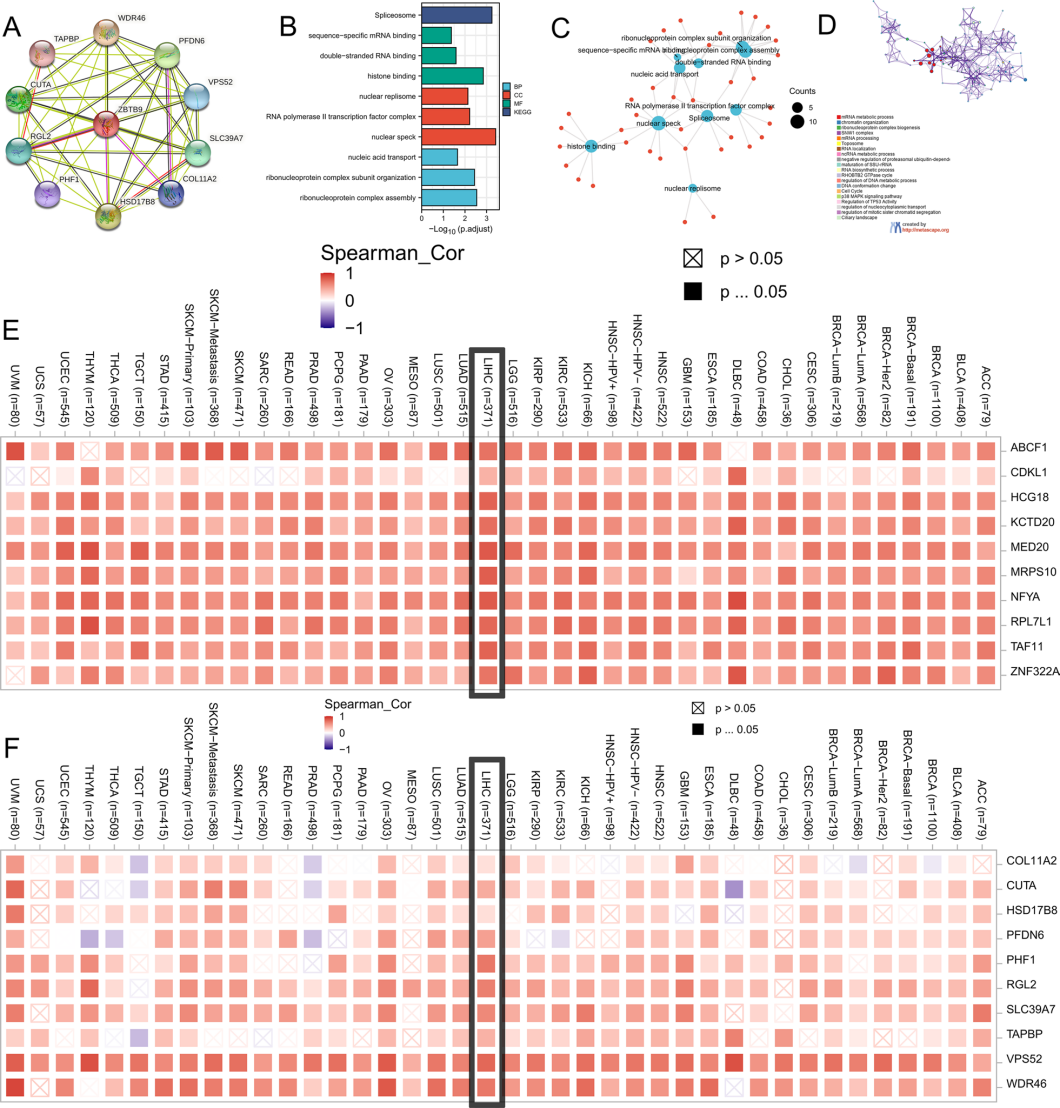
Related genes and functional enrichment analysis of ZBTB9. PPI analysis of ZBTB9 protein with STING tool (**A**). GO/KEGG analysis of ZBTB9 associated top 50 genes (**B**, **C**). Network of GO enriched terms colored by cluster-ID, where nodes that share the same cluster-ID are typically close to each other (**D**). The expression heatmap for the top 10 in 50 genes with TIMER2 tool in cancers (**E**). The expression scatter plots for 10 in 50 genes from GEPIA2 analysis with the TIMER2 tool in LIHC (**F**)

Using the STRING tool, ZBTB9 binding proteins were selected, which were verified in before studies, there were 10 ZBTB9 binding proteins, WDR46, PFDN6, VPS52, SLC39A7, COL11A2, HSD17B8, PHF1, RGL2, CUTA, TAPBP (Fig. 8A). Additionally, we also obtained top 50 genes by the GEPIA2 tool which were positively correlated with ZBTB9 expression in LIHC.

Then the GO/KEGG analysis was performed with the above top ZBTB9 co-expressed 50 genes. The results calculated by R software were provided in Fig. 8B, C, which indicated that ZBTB9-related genes were probably associated with the process of the spliceosome, sequence-specific mRNA binding, double-stranded RNA binding, histone binding, nuclear replisome and RNA polymerase II transcription factor, which all participates in cell proliferation. In addition, the results of Metascape also resembled the above findings, suggesting that the 50 genes were related to the mRNA metabolic process and chromatin organization ribonucleoprotein complex biogenesis (Fig. 8D).

Furthermore, the top related 10 genes of the results of GEPIA2 and the 10 genes of PPI result were utilized to perform the analysis of co-expression with ZBTB9 among cancers via TIMER2. Results were visualized via heatmap (Fig. 8E, F) which showed that the 20 genes were mostly co-expressed with ZBTB9 among the bulk of cancers.

### Prognostic value analysis of ZBTB9-related genes

**Fig. 9:**
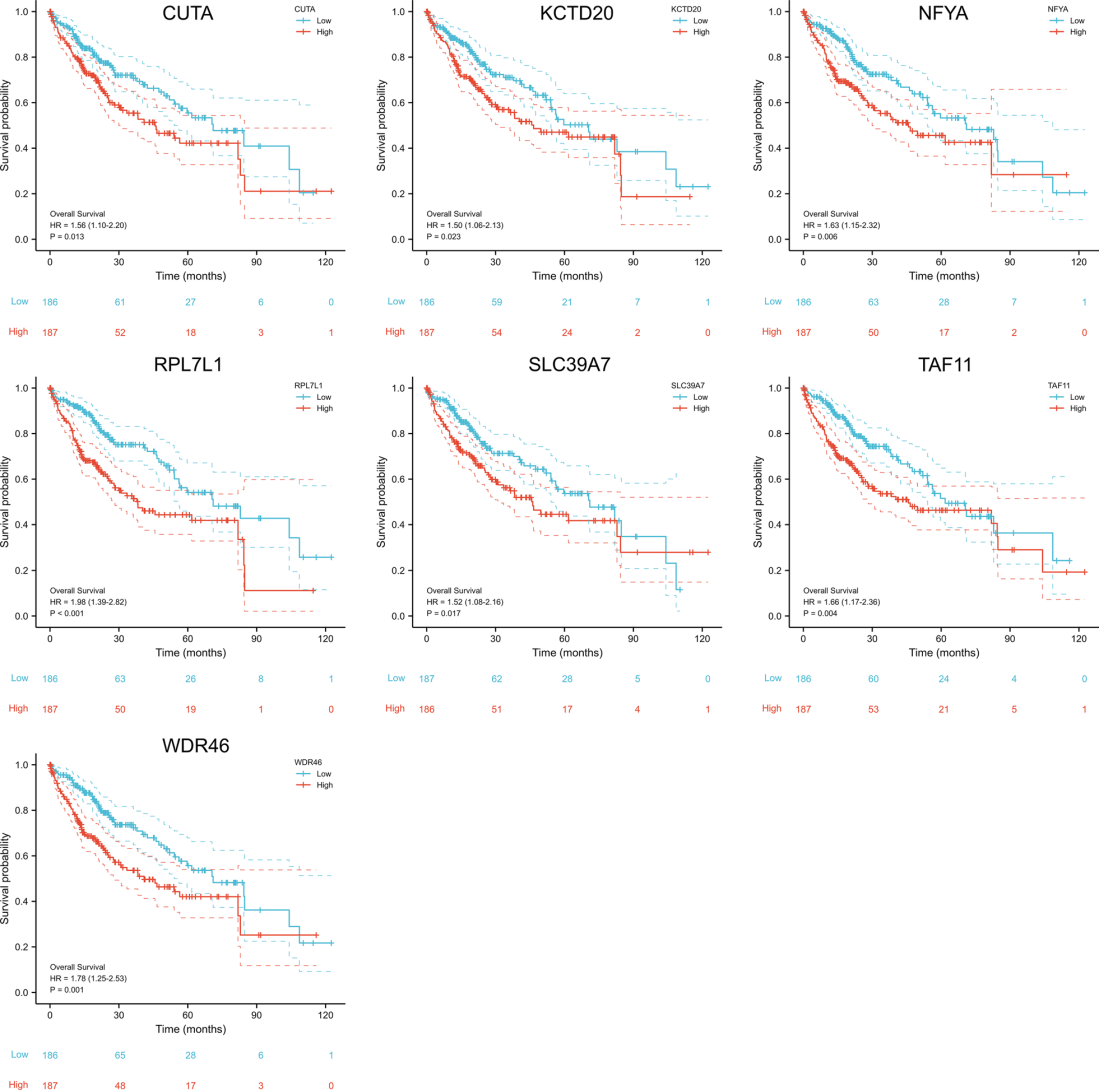
ZBTB9 related 7 genes prognostic and immunity-relation analyses. The Kaplan–Meier survival curves of CUTA (**A**). KCTD20 (**B**), NFYA (**C**), RPL7L1 (**D**), SLC39A7 (**E**), TAF11 (**F**), WDR46 (**G**)

There were 7 in the above 20 genes that showed positive results according to the results of Kaplan–Meier survival curve analyses, CUTA (HR = 1.56, 95% CI 1.10–2.20, P = 0.013), KCTD20 (HR = 1.50, 95% CI 1.06–2.13, P = 0.023), NFYA (HR = 1.63, 95% CI 1.15–2.32, P = 0.006), RPL7L1 (HR = 1.98, 95% CI 1.39–2.82, P < 0.001), SLC39A7 (HR = 1.52, 95% CI 1.08–2.16, P = 0.017), TAF11 (HR = 1.66, 95% CI 1.17–2.36, P = 0.004), WDR46 (HR = 1.78 95% CI 1.25–2.53, P = 0.001) (Fig. 9A–G). These analyses of ZBTB9-related genes probably suggested that these molecules together contributed to tumor progression.

### Function analysis of top-related genes

**Fig. 10:**
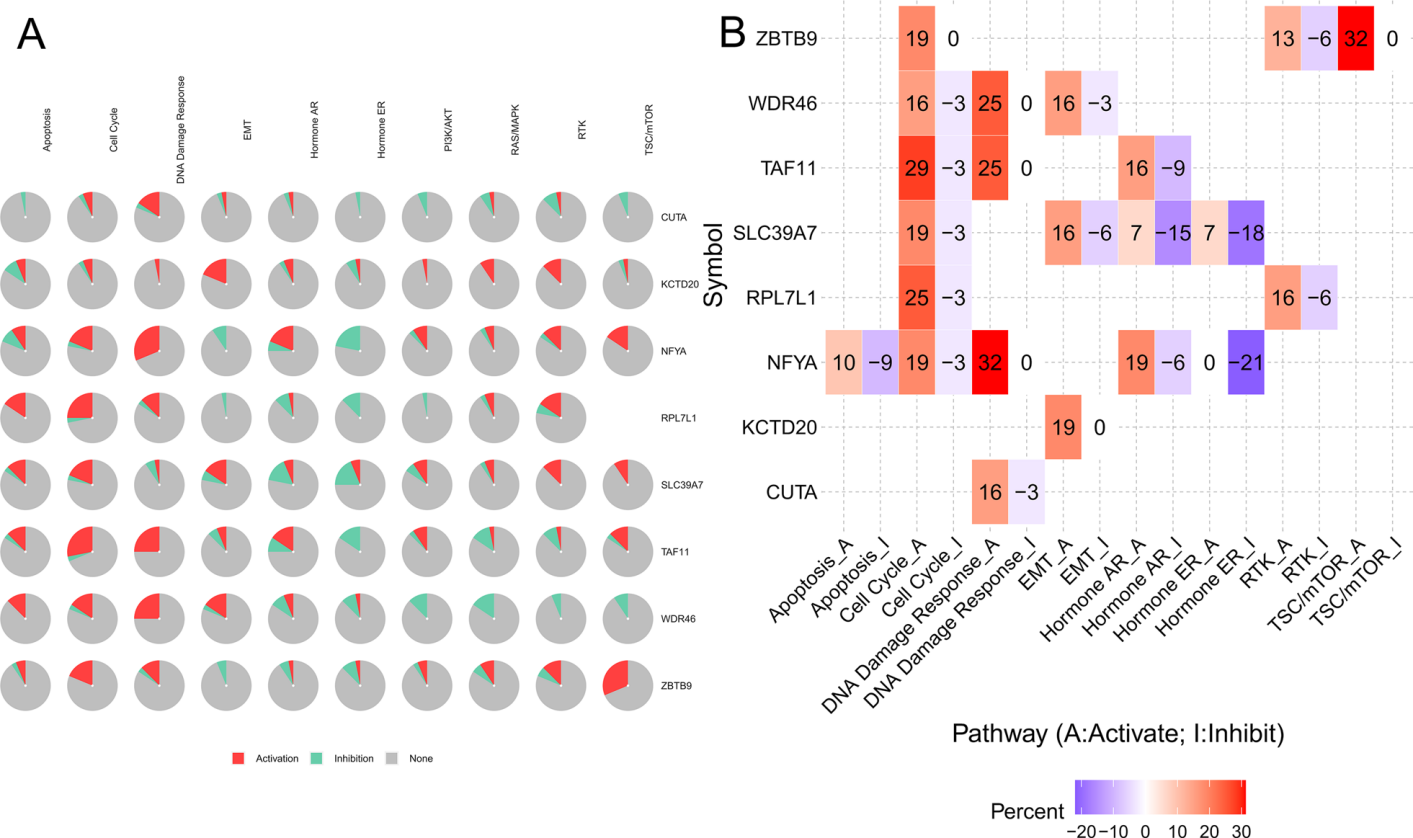
function analysis of 8 genes in LIHC. Signaling pathways analysis of 8 genes (**A**, **B**), indicating that these genes could mainly trigger the cell cycle pathways activation, and DNA repair

With GSCALite (https://www.editorialmanager.com/jtrm/default1.aspx) [31] tool, we analyzed the together function of the above 7 genes and ZBTB9 in LIHC, which indicated that these genes could mainly trigger the cell cycle pathways activation, and DNA repair, then resulted in tumor progression. (Fig. 10A, B).

### Validation with in vitro experiments

Given the above multi-aspects findings, ZBTB9 was an oncogene driving tumor initiation and progression in LIHC, which could lead to poor prognosis via activating cell proliferation and rendering immune dysfunction. We then performed a series of experiments to verify whether the malignant biological behaviors could be inhibited via the knockdown of ZBTB9 at the cell level.

**Fig. 11:**
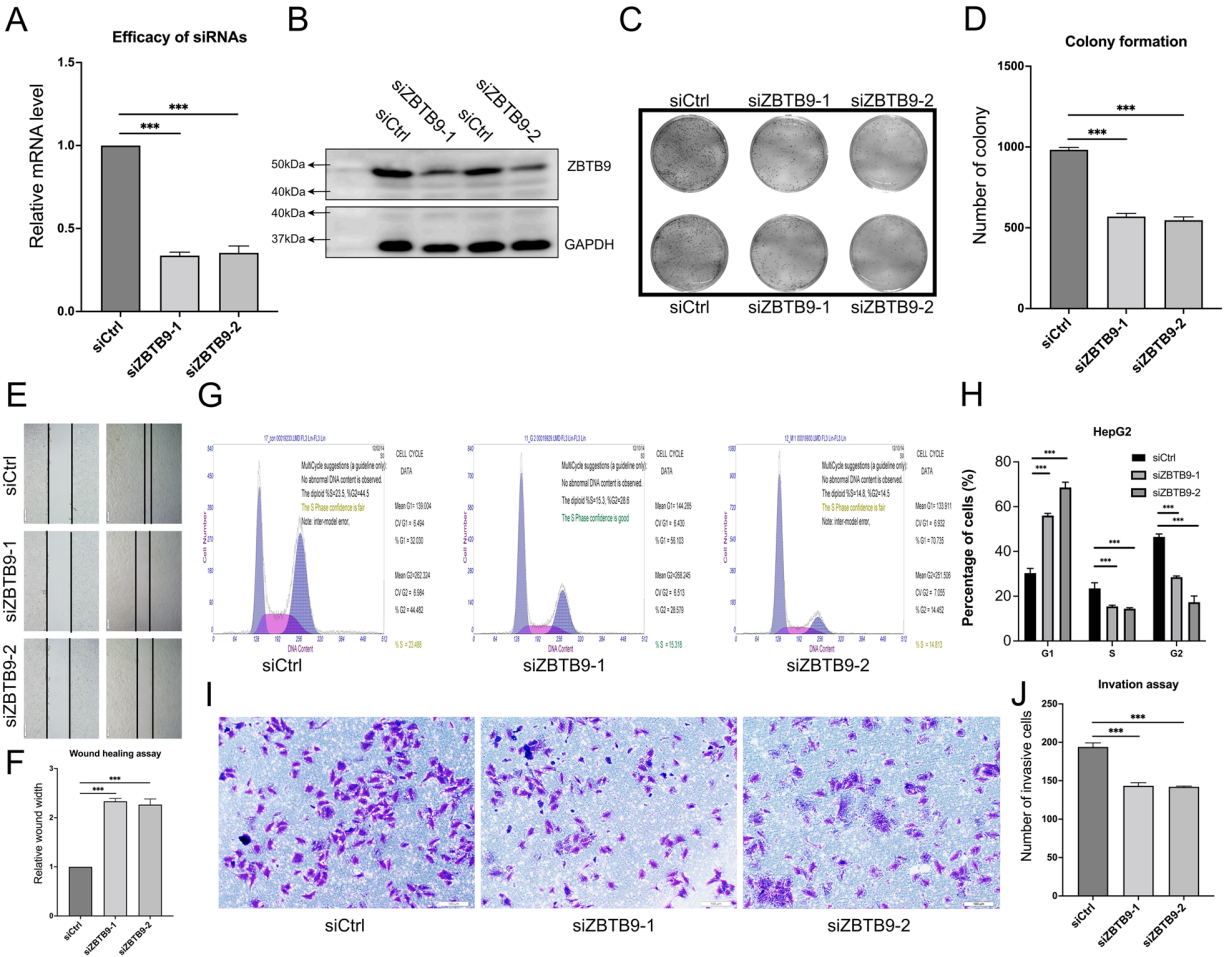
ZBTB9 knockdown blocks the proliferation and metastasis in vitro. The evaluation for efficiency of siRNA via RT-qPCR (**A**) and WB (**B**). Colony assay showed the number of ZBTB9 knockdown HepG2 cells was significantly less than siCtrl group cells (**C**, **D**). Wound healing assay showed the wound widths of ZBTB9 knockdown groups were significantly wider than the siCtrl group, after 16 h (200 μm) (**E**, **F**). With the analysis of the HepG2 cell cycle by flow cytometry, results showed cells in ZBTB9 knockdown groups were significantly stuck in the G1 phase (**G**, **H**). Results of the cells transwell assay showed that the migratory cells of ZBTB9 knockdown groups were significantly less than siCtrl groups (100 μm) (**I**, **J**). Every experiment was conducted with at least three biological replications (*P < 0.05; **P < 0.01; ***P < 0.001)

Firstly, the knockdown efficiency of siZBTB9-1 and siZBTB9-2 was evaluated by RT-qPCR, which showed that the siRNAs reduced the mRNA level of ZBTB9 in HepG2 cells (Fig. 11A), and the subsequent WB assay also verified it (Fig. 11B, and its whole result was provided at Additional file 3: Fig. S3).

The effects of ZBTB9 knockdown on cell proliferation were examined via colony formation assay. Compared to the siCtrl group, the number of HepG2 colonies groups was significantly reduced in the ZBTB9 knockdown groups (Fig. 11C, D, P < 0.01). Results of the wound healing assay also indicated that compared to the siCtrl group, wider wounds after the same interval of 16 h were observed in ZBTB9 knockdown groups (Fig. 11E, F).

Flow cytometry analysis was performed to analyze the impacts of ZBTB9 knockdown on the cell cycle. The results suggested that the cell number at the G1 phase of the two ZBTB9 knockdown groups was significantly increased compared to the siCtrl group, and the cell number in the S and G2 phases was decreased (Fig. 11G, H, P < 0.001), suggesting that via blocking ZBTB9 in HepG2, cells the proliferation capacity was evident impaired.

Furthermore, the effects of ZBTB9 knockdown on cell invasion were assessed using the transwell assay, which showed that ZBTB9 knockdown significantly reduced the invasive ability of HepG2 cells (Fig. 11I, J, P < 0.001).

The conclusions from the above experiments demonstrated that after the knockdown of ZBTB9 in HepG2 cells, the abilities of cell proliferation and migration were evidently inhibited, which strongly confirmed our previous results.

## Discussion

LIHC is still one of the most aggressive cancers therefore a major public health challenge, which deserves more investigations for effective biomarkers and therapeutic targets. Here, we first investigated the role of the ZBTB9 gene in LIHC.

With the results of related analyses, we found that ZBTB9 was overexpressed in many types of tumors, including LIHC (P < 0.001), and two LIHC cohorts’ data from GEO also proved its abnormal expression. In addition, the levels of ZBTB9 showed the relation with higher tumor grade and individual stages for LIHC patients. Given promoter hypomethylation of oncogenes has been considered as a tumor initiation and development promotive factor, it could result in gene overexpression, and downstream signaling pathways activation [32, 33], our related results showed that ZBTB9 promoter methylation was evidently lower in tumor tissues compared to adjacent normal tissues, and the genomic alteration was another potential reason for its upregulation.

Survival analysis was conducted to evaluate the prognostic value of ZBTB9, which indicated that LIHC patients who had high ZBTB9 expression tended to possess shorter OS (HR = 1.85, 95% CI 1.29–2.64, P < 0.001) and DSS (HR = 2.14, 95% CI 1.34–3.43, P < 0.001) based on TCGA data. Similarly, the survival analysis results of the tissue microarray further confirmed that ZBTB9 was also an evidently risk factor for LIHC patients OS. In addition, the Cox regression analysis based on pan-cancer data also demonstrated that ZBTB9 was a notable risk factor in many cancers, including LIHC. These findings all indicated that ZBTB9 was not only an overexpressed gene but a significant risk factor for the prognosis of LIHC patients. Genomic alteration and promoter hypomethylation could be the etiology for its abnormal expression. Furthermore, the correlation analysis between the efficacy of clinical drugs and ZBTB9 expression showed that there were 9 in 11 significantly negative correlations with drug efficacy (Cor < 0, P < 0.05), suggesting the value of ZBTB9 in the guidance for LIHC patients’ precise treatments.

Given the above findings, we conducted a series of analyses to investigate the downstream mechanisms for its carcinogenetic and risk role. It has been reported that the characteristics of TIME play a crucial role in LIHC initiation and progression [34, 35], therefore, the relationship between ZBTB9 and TIME was analyzed, subsequently showing that ZBTB9 had negative relationships with the infiltration of lymphocytes, (Th1, CD8+ T cells), immune checkpoint stimulators (IL6, IL2R) and immune checkpoint inhibitors (CD274, CTLA-4). The correlations between ZBTB9, CD8A, and FOXP3 were preliminarily verified with IHC analysis based on LIHC samples, which showed that high ZBTB9 levels in LIHC patients might be associated with the lower infiltration of CD8+ T cells and Tregs (although the IHC result of CD8A in sample #2 was positive too, the proportion of high positive intensity was still lower than sample #1). These findings indicated that the high expression of ZBTB9 might lead to a “COLD” TIME and be related to a low response to immunotherapy.

Further GSEA analysis showed that in high ZBTB9 expression samples, the G2M checkpoint, epithelial-mesenchymal transition, E2F targets, and KRAS-related signaling pathways were significantly activated, which has been reported to participate in tumor proliferation and progression [36, 37]. In addition, the investigation of the correlation between ZBTB9 and KRAS signaling pathway demonstrated that ZBTB9 was evidently associated with MAPK1, NRAS, SHP2, SOS1, and KRAS2 (all play imperative roles in KRAS pathway [38–40]), suggesting that the malignant biological behaviors under ZBTB9 upregulation were probably mediating through this pathways. Blocking of ZBTB9 could have the potential value to optimize the efficacy of inhibitors against KRAS signaling pathways [41, 42].

Similarly, based on ZBTB9-related genes, the results of correlated biological functions and signaling pathways analysis indicated that these genes participated in triggering cell cycle activation, which suggested that these genes possessed synergy effects on the promotion of tumor initiation and development at some extent.

To credibly identify the role of ZBTB9 in LIHC, we further conducted a series of cell experiments. After ZBTB9 knockdown, the cell cycles of HepG2 cells were mainly stuck in the G1 phase with P < 0.001 and the invasion assay showed that after the suppression of ZBTB9, the cell competence of migration was significantly impaired (P < 0.001). In addition, after the same time interval, wound healing assay and colony assay indicated that a wider wound and a less number of colonies were detected in the ZBTB9 knockdown groups compared to siCtrl groups. Which confirmed the promotive role of ZBTB9 in tumor cell development.

It needs to be acknowledged that there are limitations of our study. The clear downstream molecule of ZBTB9 expression was not comprehensively identified with current experiments and the limited number of LIHC samples. More rigorous in vivo experiments also need to be conducted to confirm the current findings and help to reveal novel characteristics for ZBTB9 role in LIHC, and it is also what we will further explore in the future.

This study is based on the evidence from multi-omics, experiments, and clinical samples to identify that ZBTB9, a member of ZBTB proteins, could promote tumor proliferation and migration, which possesses the strong potential to be a novel biomarker and to offer new treatment targets for LIHC patients.

## Supplementary Information

**Additional file 1: Figure S1.** Results analysis of the MSP agarose gel electrophoresis. M labels represent the results of promoter methylation, and U labels represent the results of non-promoter methylation. The odd labels represent adjacent normal tissues and even labels represent tumor tissues (A). The red dots and blues dots represent the quantitative results of tumor and adjacent normal tissues, and the green dots in the right part represent the difference value (paired adjacent normal tissue—tumor tissue) between the quantitative results of paired adjacent normal tissues and tumor tissues, with pixels as scales. The result based on paired T-test showed that ZBTB9 promoter methylation was evidently lower in tumor tissues than the paired adjacent normal tissues (B, P = 0.03).

**Additional file 2: Figure S2.** Correlation between ZBTB9 level and CD8A, FOXP3 in TIME (50 μm). Tumor tissues were obtained from two LIHC patients, who had different expression level of ZBTB9, marked as sample #1 and sample #2. The blue dots represent cell nuclear, and the brown or yellow stainings represent the target proteins. According to the intensity and the comparison of the number of brown or yellow stainings and blue dots, the relatively quantitation of protein expression could be observed. In the relatively high ZBTB9 level patient (sample #2), the relatively low levels of CD8A (D) and FOXP3 (F) were observed.

**Additional file 3: Figure S3.** The efficacy of siRNAs was detected via WB assay. The groups contained the samples of siCtrl, siZBTB9-1 and siZBTB9-2, and two target proteins, ZBTB9 and GAPDH. Results showed that the expression levels of ZBTB9 were significantly downregulated after inhibition.

## Data Availability

Bioinformatics datasets presented in this study can be found in online repositories, and the datasets used and/or analyzed during experiments are available from the corresponding author on reasonable request.

## References

[1] Torre LA, Sung H, Ferlay J, Siegel RL, Laversanne M, Soerjomataram I (2021). Global cancer statistics, 2020. CA Cancer J Clin.

[2] Ye QH, Zhu WW, Zhang JB, Qin Y, Lu M, Lin GL, Guo L, Zhang B, Lin ZH, Roessler S (2016). GOLM1 modulates EGFR/RTK cell-surface recycling to drive hepatocellular carcinoma metastasis. Cancer Cell.

[3] Zhou X, Wen Y, Tian Y, He M, Ke X, Huang Z, He Y, Liu L, Scharf A, Lu M (2019). Heat shock protein 90α-dependent B-Cell-2-associated transcription factor 1 promotes hepatocellular carcinoma proliferation by regulating MYC proto-oncogene c-MYC mRNA stability. Hepatology.

[4] Kelly KF, Daniel JM (2006). POZ for effect–POZ-ZF transcription factors in cancer and development. Trends Cell Biol.

[5] Lee SU, Maeda T (2012). POK/ZBTB proteins: an emerging family of proteins that regulate lymphoid development and function. Immunol Rev.

[6] Xiang T, Tang J, Li L, Peng W, Du Z, Wang X, Li Q, Xu H, Xiong L, Xu C (2019). Tumor suppressive BTB/POZ zinc-finger protein ZBTB28 inhibits oncogenic BCL6/ZBTB27 signaling to maintain p53 transcription in multiple carcinogenesis. Theranostics.

[7] Ahmed S, Khan S, Qureshi MA, Bukhari U, Anis M, Mughal MN (2022). Expressional variations of Kaiso: an association with pathological characteristics and field cancerization of OSCC. BMC Cancer.

[8] Wang Z, Zhao X, Wang W, Liu Y, Li Y, Gao J, Wang C, Zhou M, Liu R, Xu G, Zhou Q (2018). ZBTB7 evokes 5-fluorouracil resistance in colorectal cancer through the NF-κB signaling pathway. Int J Oncol.

[9] To JC, Chiu AP, Tschida BR, Lo LH, Chiu CH, Li XX, Kuka TP, Linden MA, Amin K, Chan WC (2021). ZBTB20 regulates WNT/CTNNB1 signalling pathway by suppressing PPARG during hepatocellular carcinoma tumourigenesis. JHEP Rep.

[10] Peterson ML, Ma C, Spear BT (2011). Zhx2 and Zbtb20: novel regulators of postnatal alpha-fetoprotein repression and their potential role in gene reactivation during liver cancer. Semin Cancer Biol.

[11] Li T, Fu J, Zeng Z, Cohen D, Li J, Chen Q, Li B, Liu XS (2020). TIMER2.0 for analysis of tumor-infiltrating immune cells. Nucleic Acids Res.

[12] Liu J, Lichtenberg T, Hoadley KA, Poisson LM, Lazar AJ, Cherniack AD, Kovatich AJ, Benz CC, Levine DA, Lee AV (2018). An integrated TCGA pan-cancer clinical data resource to drive high-quality survival outcome analytics. Cell.

[13] Goldman MJ, Craft B, Hastie M, Repečka K, McDade F, Kamath A, Banerjee A, Luo Y, Rogers D, Brooks AN (2020). Visualizing and interpreting cancer genomics data via the Xena platform. Nat Biotechnol.

[14] Tang Z, Kang B, Li C, Chen T, Zhang Z (2019). GEPIA2: an enhanced web server for large-scale expression profiling and interactive analysis. Nucleic Acids Res.

[15] Maclean A, Bunni E, Makrydima S, Withington A, Kamal AM, Valentijn AJ, Hapangama DK (2020). Fallopian tube epithelial cells express androgen receptor and have a distinct hormonal responsiveness when compared with endometrial epithelium. Hum Reprod.

[16] Dogan S, Vasudevaraja V, Xu B, Serrano J, Ptashkin RN, Jung HJ, Chiang S, Jungbluth AA, Cohen MA, Ganly I (2019). DNA methylation-based classification of sinonasal undifferentiated carcinoma. Mod Pathol.

[17] Sheehan B, Neeb A, Buroni L, Paschalis A, Riisnaes R, Gurel B, Gil V, Miranda S, Crespo M, Guo C (2022). Prostate-specific membrane antigen expression and response to DNA damaging agents in prostate cancer. Clin Cancer Res.

[18] Schneider CA, Rasband WS, Eliceiri KW (2012). NIH Image to ImageJ: 25 years of image analysis. Nat Methods.

[19] Varghese F, Bukhari AB, Malhotra R, De A (2014). IHC Profiler: an open source plugin for the quantitative evaluation and automated scoring of immunohistochemistry images of human tissue samples. PLoS ONE.

[20] Cerami E, Gao J, Dogrusoz U, Gross BE, Sumer SO, Aksoy BA, Jacobsen A, Byrne CJ, Heuer ML, Larsson E (2012). The cBio cancer genomics portal: an open platform for exploring multidimensional cancer genomics data. Cancer Discov.

[21] Gao J, Aksoy BA, Dogrusoz U, Dresdner G, Gross B, Sumer SO, Sun Y, Jacobsen A, Sinha R, Larsson E (2013). Integrative analysis of complex cancer genomics and clinical profiles using the cBioPortal. Sci Signal.

[22] Forbes SA, Tang G, Bindal N, Bamford S, Dawson E, Cole C, Kok CY, Jia M, Ewing R, Menzies A (2010). COSMIC (the Catalogue of Somatic Mutations in Cancer): a resource to investigate acquired mutations in human cancer. Nucleic Acids Res.

[23] Chandrashekar DS, Bashel B, Balasubramanya SAH, Creighton CJ, Ponce-Rodriguez I, Chakravarthi B, Varambally S (2017). UALCAN: a portal for facilitating tumor subgroup gene expression and survival analyses. Neoplasia.

[24] Reinhold WC, Sunshine M, Liu H, Varma S, Kohn KW, Morris J, Doroshow J, Pommier Y (2012). Cell Miner: a web-based suite of genomic and pharmacologic tools to explore transcript and drug patterns in the NCI-60 cell line set. Cancer Res.

[25] Shankavaram UT, Varma S, Kane D, Sunshine M, Chary KK, Reinhold WC, Pommier Y, Weinstein JN (2009). Cell Miner: a relational database and query tool for the NCI-60 cancer cell lines. BMC Genomics.

[26] Ru B, Wong CN, Tong Y, Zhong JY, Zhong SSW, Wu WC, Chu KC, Wong CY, Lau CY, Chen I (2019). TISIDB: an integrated repository portal for tumor-immune system interactions. Bioinformatics.

[27] Subramanian A, Tamayo P, Mootha VK, Mukherjee S, Ebert BL, Gillette MA, Paulovich A, Pomeroy SL, Golub TR, Lander ES, Mesirov JP (2005). Gene set enrichment analysis: a knowledge-based approach for interpreting genome-wide expression profiles. Proc Natl Acad Sci USA.

[28] Yu G, Wang LG, Han Y, He QY (2012). clusterProfiler: an R package for comparing biological themes among gene clusters. OMICS.

[29] Liberzon A, Subramanian A, Pinchback R, Thorvaldsdóttir H, Tamayo P, Mesirov JP (2011). Molecular signatures database (MSigDB) 3.0. Bioinformatics.

[30] Zhou Y, Zhou B, Pache L, Chang M, Khodabakhshi AH, Tanaseichuk O, Benner C, Chanda SK (2019). Metascape provides a biologist-oriented resource for the analysis of systems-level datasets. Nat Commun.

[31] Liu CJ, Hu FF, Xia MX, Han L, Zhang Q, Guo AY (2018). GSCALite: a web server for gene set cancer analysis. Bioinformatics.

[32] Lee SM, Lee YG, Bae JB, Choi JK, Tayama C, Hata K, Yun Y, Seong JK, Kim YJ (2014). HBx induces hypomethylation of distal intragenic CpG islands required for active expression of developmental regulators. Proc Natl Acad Sci U S A.

[33] Guo T, Gong C, Wu P, Battaglia-Hsu SF, Feng J, Liu P, Wang H, Guo D, Yao Y, Chen B (2020). LINC00662 promotes hepatocellular carcinoma progression via altering genomic methylation profiles. Cell Death Differ.

[34] Luo Y, Wang J, Xu L, Du Q, Fang N, Wu H, Liu F, Hu L, Xu J, Hou J (2022). A theranostic metallodrug modulates immunovascular crosstalk to combat immunosuppressive liver cancer. Acta Biomater.

[35] Yang P, Qin H, Li Y, Xiao A, Zheng E, Zeng H, Su C, Luo X, Lu Q, Liao M (2022). CD36-mediated metabolic crosstalk between tumor cells and macrophages affects liver metastasis. Nat Commun.

[36] Babaei G, Vostakolaei MA, Bazl MR, Aziz SG, Gholipour E, Nejati-Koshki K (2022). The role of exosomes in the molecular mechanisms of metastasis: focusing on EMT and cancer stem cells. Life Sci.

[37] Katoch S, Sharma V, Patial V (2022). Peroxisome proliferator-activated receptor gamma as a therapeutic target for hepatocellular carcinoma: experimental and clinical scenarios. World J Gastroenterol.

[38] Huang KL, Scott AD, Zhou DC, Wang LB, Weerasinghe A, Elmas A, Liu R, Wu Y, Wendl MC, Wyczalkowski MA (2021). Spatially interacting phosphorylation sites and mutations in cancer. Nat Commun.

[39] Mentrasti G, Cantini L, Zichi C, D'Ostilio N, Gelsomino F, Martinelli E, Chiari R, La Verde N, Bisonni R, Cognigni V (2022). Alarming drop in early stage colorectal cancer diagnoses after COVID-19 outbreak: a real-world analysis from the Italian COVID-DELAY Study. Oncologist.

[40] Kaubryte J, Lai AG (2022). Pan-cancer prognostic genetic mutations and clinicopathological factors associated with survival outcomes: a systematic review. NPJ Precis Oncol.

[41] Song Y, Yang X, Wang S, Zhao M, Yu B (2022). Crystallographic landscape of SHP2 provides molecular insights for SHP2 targeted drug discovery. Med Res Rev.

[42] Theard PL, Sheffels E, Sealover NE, Linke AJ, Pratico DJ, Kortum RL (2020). Marked synergy by vertical inhibition of EGFR signaling in NSCLC spheroids shows SOS1 is a therapeutic target in EGFR-mutated cancer. Elife.

