# Supplementary figures and images for: Identification of ZBTB9 as a potential therapeutic target against dysregulation of tumor cells proliferation and a novel biomarker in Liver Hepatocellular Carcinoma
Source: J Transl Med. 2022 Dec 15;20:602. doi: 10.1186/s12967-022-03790-0 (PMC9756481; doi:10.1186/s12967-022-03790-0)

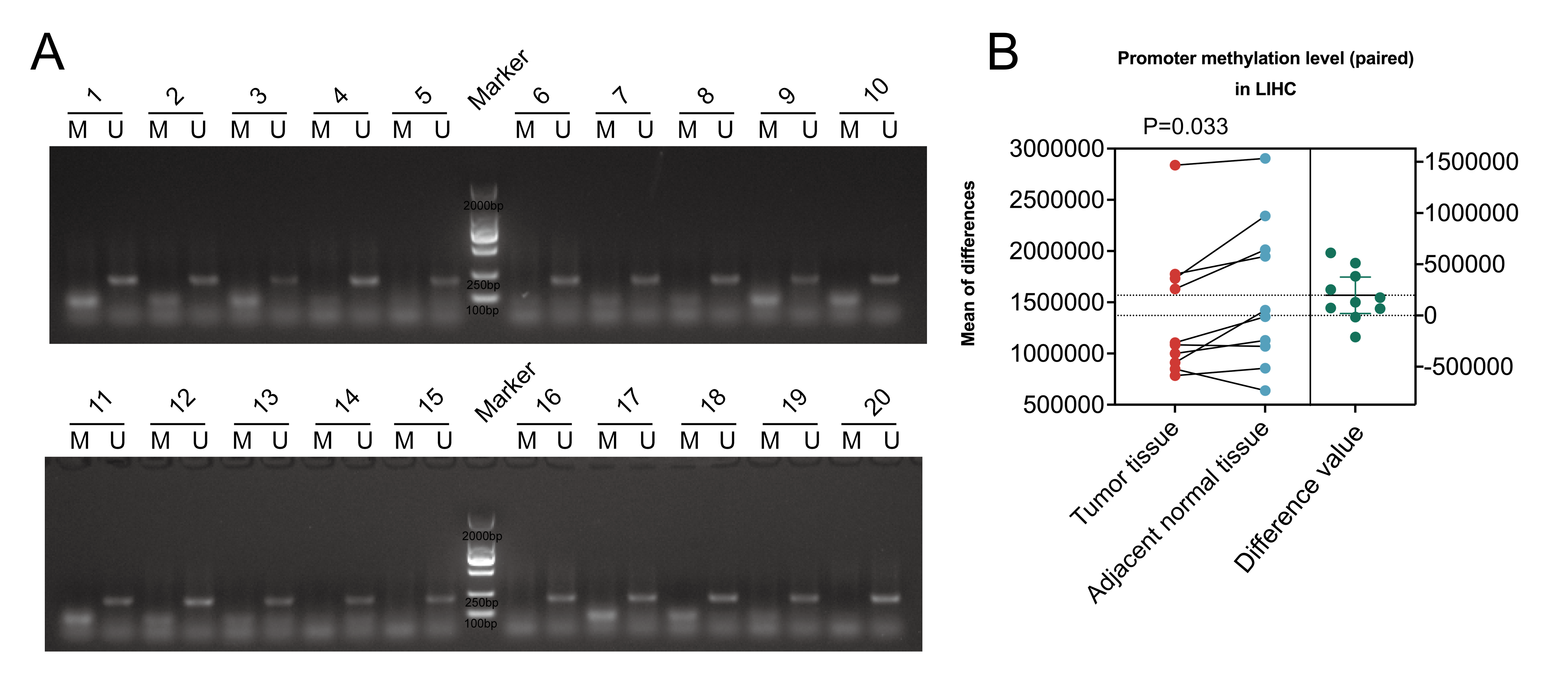

Supplement: Supplementary file 1 — Additional file 1: Figure S1. Results analysis of the MSP agarose gel electrophoresis. M labels represent the results of promoter methylation, and U labels represent the results of non-promoter methylation. The odd labels represent adjacent normal tissues and even labels represent tumor tissues (A). The red dots and blues dots represent the quantitative results of tumor and adjacent normal tissues, and the green dots in the right part represent the difference value (paired adjacent normal tissue—tumor tissue) between the quantitative results of paired adjacent normal tissues and tumor tissues, with pixels as scales. The result based on paired T-test showed that ZBTB9 promoter methylation was evidently lower in tumor tissues than the paired adjacent normal tissues (B, P = 0.03). [file 12967_2022_3790_MOESM1_ESM.png]

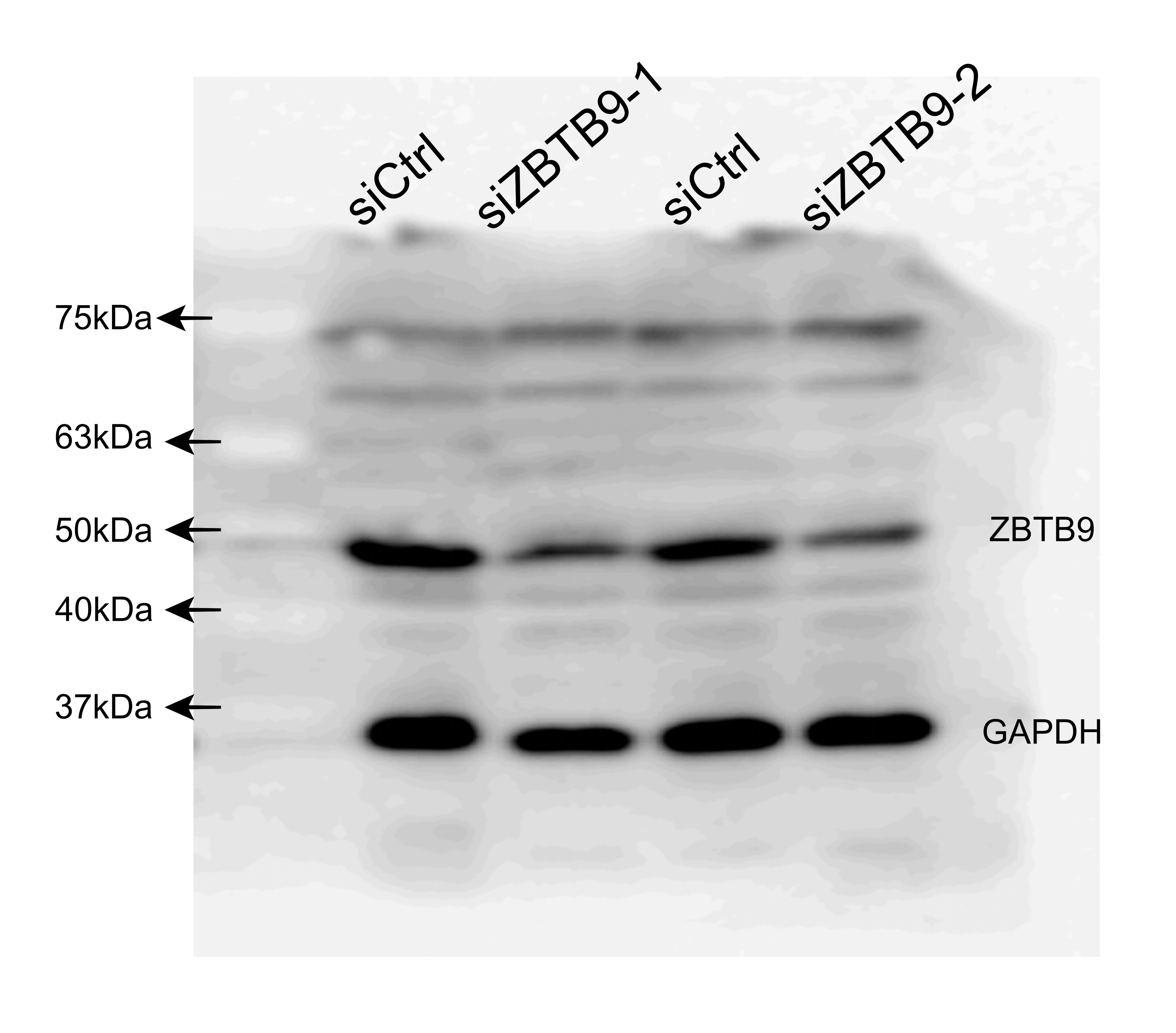

Supplement: Supplementary file 3 — Additional file 3: Figure S3. The efficacy of siRNAs was detected via WB assay. The groups contained the samples of siCtrl, siZBTB9-1 and siZBTB9-2, and two target proteins, ZBTB9 and GAPDH. Results showed that the expression levels of ZBTB9 were significantly downregulated after inhibition. [file 12967_2022_3790_MOESM3_ESM.png]
